# Individual retrotransposon integrants are differentially controlled by KZFP/KAP1-dependent histone methylation, DNA methylation and TET-mediated hydroxymethylation in naïve embryonic stem cells

**DOI:** 10.1186/s13072-018-0177-1

**Published:** 2018-02-26

**Authors:** Andrea Coluccio, Gabriela Ecco, Julien Duc, Sandra Offner, Priscilla Turelli, Didier Trono

**Affiliations:** School of Life Sciences, Ecole Polytechnique Federale de Lausanne (EPFL), Station 19, 1015 Lausanne, Switzerland

**Keywords:** KAP1, KZFPs, TET, DNA methylation, Hydroxymethylation, Genomic imprinting, Transposable elements, Epigenetic reprogramming

## Abstract

**Background:**

The KZFP/KAP1 (KRAB zinc finger proteins/KRAB-associated protein 1) system plays a central role in repressing transposable elements (TEs) and maintaining parent-of-origin DNA methylation at imprinting control regions (ICRs) during the wave of genome-wide reprogramming that precedes implantation. In naïve murine embryonic stem cells (mESCs), the genome is maintained highly hypomethylated by a combination of TET-mediated active demethylation and lack of de novo methylation, yet KAP1 is tethered by sequence-specific KZFPs to ICRs and TEs where it recruits histone and DNA methyltransferases to impose heterochromatin formation and DNA methylation.

**Results:**

Here, upon removing either KAP1 or the cognate KZFP, we observed rapid TET2-dependent accumulation of 5hmC at both ICRs and TEs. In the absence of the KZFP/KAP1 complex, ICRs lost heterochromatic histone marks and underwent both active and passive DNA demethylation. For KAP1-bound TEs, 5mC hydroxylation correlated with transcriptional reactivation. Using RNA-seq, we further compared the expression profiles of TEs upon *Kap1* removal in wild-type, *Dnmt* and *Tet* triple knockout mESCs. While we found that KAP1 represents the main effector of TEs repression in all three settings, we could additionally identify specific groups of TEs further controlled by DNA methylation. Furthermore, we observed that in the absence of TET proteins, activation upon *Kap1* depletion was blunted for some TE integrants and increased for others.

**Conclusions:**

Our results indicate that the KZFP/KAP1 complex maintains heterochromatin and DNA methylation at ICRs and TEs in naïve embryonic stem cells partly by protecting these loci from TET-mediated demethylation. Our study further unveils an unsuspected level of complexity in the transcriptional control of the endovirome by demonstrating often integrant-specific differential influences of histone-based heterochromatin modifications, DNA methylation and 5mC oxidation in regulating TEs expression.

**Electronic supplementary material:**

The online version of this article (10.1186/s13072-018-0177-1) contains supplementary material, which is available to authorized users.

## Background

KAP1 (KRAB-associated protein 1), also known as tripartite-motif containing protein 28 (TRIM28), is the central component of a transcriptional repressor complex encompassing histone methylation, histone deacetylation, DNA methylation and chromatin remodelling activities [1]. KAP1 is recruited to particular genomic loci by KRAB-containing zinc finger proteins (KZFPs), a large family of rapidly evolving sequence-specific DNA-binding factors [2–6]. It then serves as a scaffold for a macromolecular complex that induces notably the di- and tri-methylation of histone 3 and DNA methylation on CpG dinucleotides, generating a highly heterochromatic environment characterized by the presence of the H3K9me2/3 repressive mark and the presence of 5mC (5-methylcytosine) [1]. The KZFP/KAP1 complex is of paramount importance in embryonic stem cells (ESCs), where it maintains heterochromatin at imprinting control regions (ICRs) [7–9] and transposable elements (TEs) [10]. *Kap1* deletion is rapidly lethal in ESCs [10], and *Kap1* knockout murine embryos die before gastrulation [11].

ICRs are genomic loci that control in *cis* the monoallelic, parent-of-origin specific expression of imprinted genes in placental mammals [12, 13]. Imprinting is established at ICRs during gametogenesis by differential DNA methylation of paternal and maternal alleles, with patterns that are preserved in the zygote and throughout development only to be erased in primordial germ cells [14, 15]. Loss of DNA methylation at ICRs leads in human to severe growth-related or neuro-developmental imprinting disorders such as transient neonatal diabetes, Beckwith–Wiedemann, Silver–Russell, Angelman or Prader–Willi syndromes, as well as cases of molar pregnancy and infertility by oligospermia [16, 17]. ICRs from both human and mouse contain the sequence TGCCGC, often in several copies, and the methylated allele of this hexanucleotide is recognized in both species by the KZFP ZFP57, which recruits the KAP1 complex to maintain histone and DNA methylation at ICRs during preimplantation development, as demonstrated in the mouse [7, 8, 18, 19]. In human, mutations in *ZFP57* are responsible for transient neonatal diabetes [20].

The human and mouse genomes contain approximately 5 million readily identifiable inserts derived from transposable elements [21, 22], a large majority of which are endogenous retroelements, whether ERV (endogenous retroviruses), LINE and SINE (long and short interspersed nuclear elements, respectively) or, in human, the primate-specific SVA (reviewed in Friedli and Trono [23]). While most components of this endovirome are no longer transposition-competent due to the accumulation of mutations, notably in human, many can still influence gene expression through a variety of transcriptional and post-transcriptional effects (reviewed in Chuong et al. [24]). Collectively, transposable elements are major drivers of genomic evolution tightly controlled through epigenetic mechanisms exerted from the earliest stages of embryonic development, from small RNA-mediated silencing to heterochromatin formation and DNA methylation induced by the KZFP/KAP1 complex [6, 10, 25–27].

Emerging evidence indicates that the KZFP/KAP1 system, rather than inducing the permanent silencing of TEs, allows for an exquisite regulation of their transcriptional influences in developing and adult tissues [5, 6, 28–30]. In contrast, the heterochromatic and DNA methylation status of ICRs does not appear to fluctuate significantly once established. However, the mammalian genome undergoes profound chromatin remodelling through the two main waves of epigenetic reprogramming that occur during germ cell formation and right after fertilization [15, 31–34]. The latter is characterized by a general loss of DNA and H3K9 methylation, a phenomenon required to achieve pluripotency [35]. Genome demethylation occurs during this period through both a failure to re-methylate the cytosine residues of CpG dinucleotides in daughter strands produced by DNA replication, a function normally accomplished by the maintenance DNA methyltransferase DNMT1, and, at least at specific loci, active demethylation by TET proteins, which compete with de novo methyltransferases (DNMT3A, DNMT3B and their cofactor DNMT3L) to preserve a highly hypomethylated state [33, 36–38]. TET proteins (TET1, TET2 and TET3) catalyse iterative oxidation of 5-methylcytosine (5mC) to 5-hydroxymethylcytosine (5hmC) and downstream oxidative products, before an unmodified cytosine can be restored by TDG-dependent repair mechanisms [39].

The DNA methylation profile of TEs evolves rapidly during early embryogenesis, exhibiting highly dynamic patterns varying for different TE subsets, with some modulation by the genomic location of individual integrants [40, 41]. For example IAPs (intra-cisternal A-particles), a group of young and highly active murine endogenous retroviruses, mostly escape reprogramming and retain a high level of DNA methylation during the entire preimplantation period [38, 41–43]. In contrast, LINEs almost completely lose DNA methylation, to see it re-established only at the time of implantation. Yet, mESCs completely devoid of DNA methylation by knockout for *Dnmt1*, *Dnmt3a* and *Dnmt3b* display little perturbation of TE expression [44], suggesting that several layers of regulation cooperate to control the activity of transposon-derived sequences, including small RNA-based mechanisms acting at a post-transcriptional level [27, 45–48].

The epigenetic and transcriptional profiles of preimplantation pluripotent cells can be recapitulated in vitro by culturing cells derived from the inner cell mass in a medium that maintains them in the so-called naïve or ground state of pluripotency [49, 50]. The genome of ESCs cultured in these conditions is kept hypomethylated by downregulation of de novo DNMTs [51–53] and impairment of the DNA methylation maintenance machinery, caused by degradation of UHRF1 and global loss of H3K9me2, which hampers recruitment of DNMT1 to hemimethylated CpGs [54]. TETs are dispensable for maintaining this hypomethylated state, although they have been shown to modulate transcription in mESCs by recruiting chromatin regulators such as OGT (*O*-linked *β*-d-*N*-acetylglucosamine transferase) [55], SIN3 repressor complex [56] and Polycomb repressive complex 2 (PRC2) [57].

Here, we explored the interplay between the KZFP/KAP1-induced histone methylation, DNA methylation and TET-mediated demethylation in determining the epigenetic status of ICRs and transposons and regulating the expression of TEs in naïve mouse ESCs. We first defined that, in this setting, the KZFP/KAP1 complex was present at almost all H3K9me3-enriched regions of the genome. These KAP1-bearing loci retained high levels of DNA methylation even in this naïve state, probably owing to a crosstalk between this regulator, the H3K9me3 chromatin mark and the DNMT maintenance machinery. Upon *Kap1* knockdown, H3K9me3 was completely lost from heterochromatic regions, and removal of the master regulator or the underlying KZFP resulted in rapid accumulation of 5hmC and loss of 5mC both at ICRs and TEs, indicating that the KZFP/KAP1 complex normally protects these loci from demethylation and TET-mediated hydroxylation. At ICRs, this led to loss of DNA methylation. At TEs, more complex and subset-specific effects were observed, revealing differential roles for KZFP/KAP1, TET proteins and DNA methylation in the control of these genetic elements.

## Results

### KAP1 maintains H3K9me3 at ICRs and TEs and protects them from TET-dependent hydroxymethylation

**Fig. 1:**
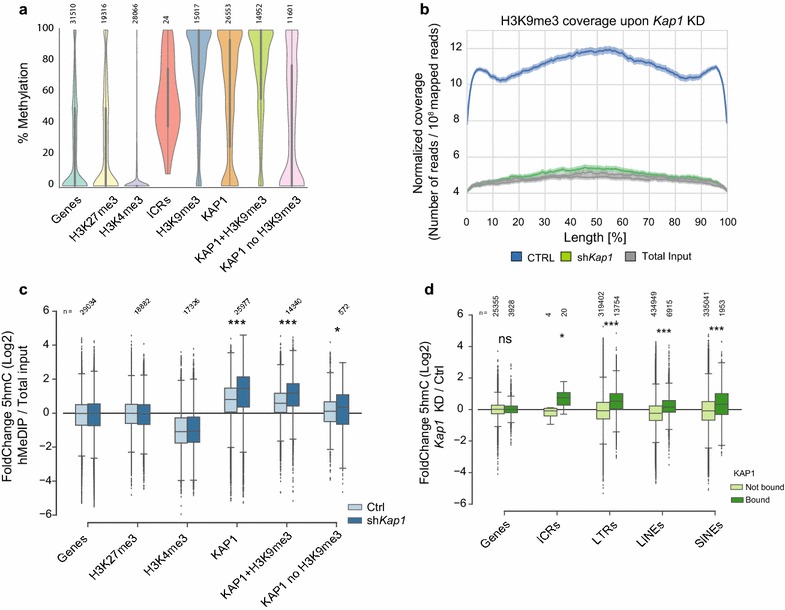
KAP1-dependent heterochromatin preserves DNA methylation in murine ESC. **a** Violin plot of the DNA methylation levels (75th percentile) measured by reduced-representation bisulfite sequencing (RRBS) on genomic regions carrying the indicated marks. **b** H3K9me3 ChIP-seq reads coverage in control and *Kap1* knockdown mESC 5 days after transduction with shRNA-expressing lentiviral vectors. Coverage is plotted over the relative length of each H3K9me3 enriched region. Data are from two independent ChIP-seq experiments. **c**, **d** Boxplot representing 5hmC enrichment over total input on selected regions measured by hMeDIP-seq (**c**) or fold change between *Kap1* knockdown and control sample (5 days post-transduction) on genomic loci either bound or not bound by KAP1 at baseline (**d**). In both cases, numbers of loci in each group are indicated on top, and statistical significance was determined by *t* test, *p < 0.05, ***p < 0.001. Data are averaged from two independent hMeDIP-seq experiments

To decipher the role of the KZFP/KAP1 complex in the maintenance of heterochromatin and DNA methylation in ground-state mESCs, we characterized the genome-wide profiles of KAP1 binding and H3K9me3 enrichment by chromatin immunoprecipitation/deep sequencing (ChIP-seq) and compared them with recently published DNA methylation data [54]. KAP1-bound loci (which cover roughly 0.9% of the genome) featured higher levels of DNA methylation compared to other genomic regions, in particular if H3K9me3 was also present (Fig. 1a). About two-thirds of H3K9me3-enriched regions overlapped with a called KAP1 peak in these cells (Additional file 1: Figure S1a). Nonetheless, *Kap1* depletion by RNA interference (Additional file 1: Figure S1b, c) resulted in a nearly complete loss of H3K9me3 at all H3K9me3 enriched regions (Fig. 1b), suggesting that KAP1 is the major driver of H3K9me3 maintenance in these cells. The loss of H3K9me3 at non-KAP1-bound loci could be either explained by the fact that these regions are false negatives and KAP1 binding occurs, but it is not detected, or because KAP1 is also part of a complex required to propagate heterochromatin during DNA replication [58, 59]. We next examined the presence of hydroxymethylated DNA, a product of TET activity, in wild-type and KAP1-depleted mESCs by hMeDIP-Seq, using antibodies against 5hmC. Genome-wide basal levels of 5hmC were expectedly low in control cells, reflecting the largely unmethylated state of the genome of naïve mESC, hence the low abundance of the 5mC precursor of 5hmC. Upon *Kap1* knockdown, regions previously bound by the regulator became enriched for 5hmC (Fig. 1c), a result confirmed by glucMS-PCR at several ICRs and at IAPEz, a subgroup of ERVs (Additional file 1: Figure S1d). Other genomic regions such as genes and loci enriched in H3K4me3 or H3K27me3 exhibited minimal changes and remained generally devoid of 5hmC. *Kap1* knockdown resulted in rapid arrest of cell proliferation and extensive cell death, starting as early as day 4 post-transduction with a *Kap1* knockdown lentiviral vector. At day 5, H3K9me3 was completely lost (Fig. 1b) and, at this time point, we could observe the most significant increase in 5hmC at KAP1 target loci (Additional file 1: Figure S1e). We subdivided ICRs and TEs according to their KAP1 binding profile and observed that only their KAP1-bound subsets became enriched in 5hmC upon KAP1 removal (Fig. 1d), correlating their higher basal degree of DNA methylation. About 20% of TEs enriched for KAP1 were targeted by hydroxymethylation, while this fraction goes up to 26% for integrants bound by KAP1 and enriched for H3K9me3. Furthermore, TE families that exhibited the highest enrichment for 5hmC such as IAPEz and MMETn were also the ones most extensively bound by KAP1 (Additional file 1: Figure S1f). Interestingly levels of 5hmC seemed higher than background on KAP1-bound loci (Fig. 1c, Additional file 1: Figure S1d, e). To test whether 5mC hydroxylation was taking place in the presence of KAP1, we performed glucMS-PCR on KAP1-bound chromatin (Additional file 2: Figure S2a, b) and found background levels of 5hmC at KAP1-bound DNA (i.e. comparable to *Tet* triple knockout cells which are devoid of the modification, Additional file 2: Figure S2a) in spite of high levels of 5mC over these regions (Additional file 2: Figure S2b). Therefore, the levels of 5hmC observed in wild-type cells might be due to stochastic loss of KAP1 at these loci in the cell population, since 5mC hydroxylation does not seem to take place at KAP1-bound chromatin. Furthermore, we performed hMeDIP followed by Sanger sequencing on mESCs from a mixed background derived from the crossing between a Cast/EiJ male and 129/Sv female (Cast/129 cells) and confirmed that 5hmC accumulates on the imprinted allele of ICRs (Additional file 2: Figure S2c). Interestingly, on H19, 5hmC was present on both alleles in the presence of KAP1, suggesting that at this locus hydroxymethlyation might be required to maintain the non-imprinted allele unmethylated.

### KZFPs and KAP1 protect their specific targets from TET-dependent and TET-independent demethylation

**Fig. 2:**
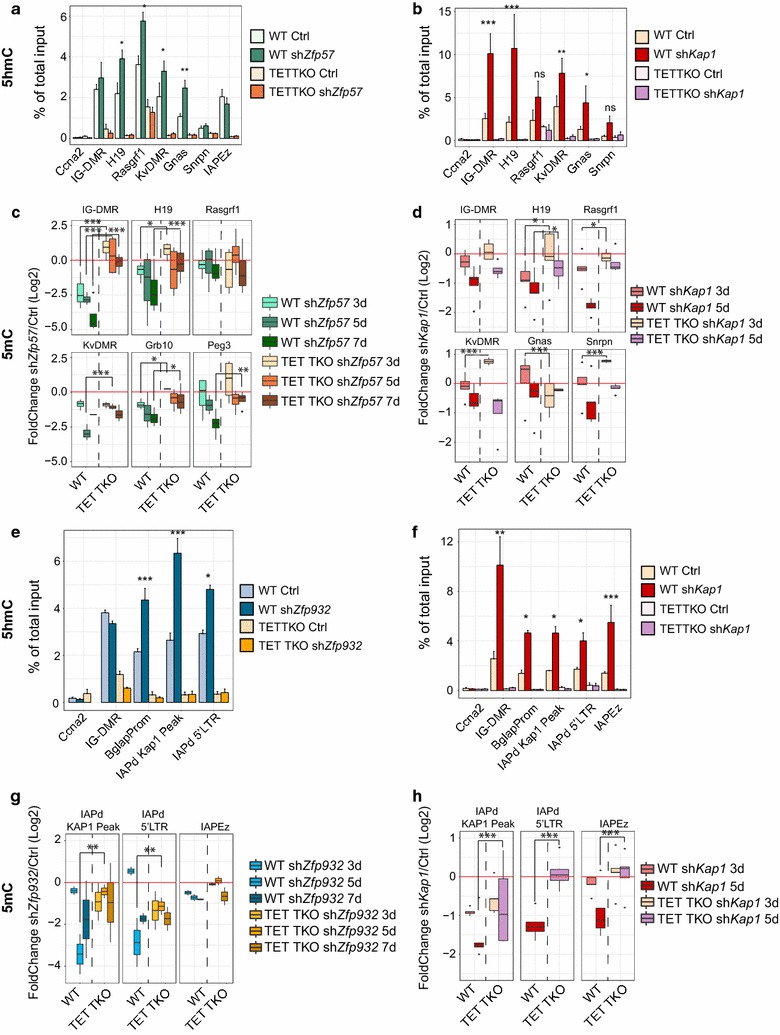
The KZFP/KAP1 complex protects ICRs and TEs from TET-mediated oxidation and loss of 5mC. **a**, **b**, **e** and **f** 5hmC enrichment measured by hMeDIP-qPCR in either wild-type or TET TKO cells upon *Kap1* (**b**, **f**), *Zfp57* (**a**) or *Zfp932* (**e**) knockdown (5 days post-transduction). Error bars represent SEM, *p < 0.05, **p < 0.01, ***p < 0.001. Student’s *t* test. *n* = 4. **c**, **d**, **g** and **h** Boxplot of 5mC enrichment, measured by MeDIP-qPCR on shown loci, at indicated times after *Kap1* (**d**, **h**), *Zfp57* (**c**) or *Zfp932* (**g**) knockdown. Data are represented as log2 of the fold change between percentages of total input for knockdown and control cells. *p < 0.05, **p < 0.01, ***p < 0.001. Student’s *t* test. *n* = 4

We then examined the dynamics of loss of 5mC upon KAP1 removal in the presence or absence of the three TET enzymes. As previously observed [10], *Kap1* knockdown resulted in arrest of cell proliferation and a wide range of transcriptional and physiological changes in mESCs rapidly leading to cell death which precluded a long-term monitoring of DNA methylation. However, since KAP1 is recruited to ICRs or TEs by sequence-specific KZFPs, we hypothesized that removing these DNA-binding proteins would expose their targets to TET-mediated hydroxymethylation and loss of 5mC. We thus used RNA interference to downregulate in mESCs either *Zfp57*, responsible for recognizing ICRs [8] or *Zfp932*, the KZFP ligand of members of the LTR/ERVK family [28]. We compared 5hmC enrichment and followed 5mC decrease in wild-type and *Tet* triple knockout (TET TKO) mESCs upon *Kap1* (Fig. 2b, d, f, h), *Zfp57* (Fig. 2a, c) and *Zfp932* (Fig. 2e, g) knockdown. Since traditional bisulfite sequencing does not discriminate between 5mC and 5hmC, we monitored 5mC levels by MeDIP. ZFP57 depletion (Additional file 3: Figure S3a) resulted in the accumulation of 5hmC at ICRs, but not at TEs such as IAPEz (Fig. 2a). We confirmed this result by glucMS-qPCR (Additional file 3: Figure S3b). We could detect an increase in 5hmC only at a few ICRs (*Gnas*, *H19*, KvDMR, *Rasgrf1* and to a lesser extent IG-DMR), while others seemed unaffected by *Zfp57* knockdown, but this phenomenon was completely abrogated in TET TKO mESC (Fig. 2a and Additional file 3: Figure S3b). Interestingly, individual ICRs behaved differently upon KAP1 or ZFP57 withdrawal, suggesting that other KRAB-ZFPs might play a role in regulation of imprinting. 5mC levels at these ICRs decreased rapidly after *Zfp57* knockdown in wild-type cells (Fig. 2c). In contrast, they dropped only slowly in their *Zfp57* knockdown TET TKO counterparts (Fig. 2c). We confirmed this enrichment results by bisulfite sequencing at day 7 after transduction (Additional file 3: Figure S3c). Thus, TET-mediated demethylation participated in the loss of DNA methylation observed at ZFP57-deprived loci. A similar tendency could be observed upon KAP1 removal (Fig. 2d), although much less pronounced, probably because passive loss of 5mC was prevented by the arrest of cell proliferation. ZFP932 binds to and regulates members of the IAP-d and MERVK10C families both in mESCs and in differentiated tissues [28]. ZFP932 notably recognizes an IAP-d element, the 3′ long terminal repeat (LTR) of which acts as promoter for the *Bglap3* gene (Additional file 3: Figure S3d) [28]. Here, we found that both *Kap1* and *Zfp932* knockdown (Additional file 3: Figure S3e) in naïve mESC induced the accumulation of 5hmC at the KAP1-binding region of the *Bglap3*-controlling IAP-d integrant and on its 3′LTR, whereas the 5′LTR was less affected (Fig. 2e, f). Depletion of either protein resulted in loss of DNA methylation both at the 5′LTR and at 3′LTR (Fig. 2g, h), and this was more pronounced in wild-type cells than in TET TKO cells, indicating that hydroxymethylation contributes to the loss of 5mC at KAP1-controlled TEs. Surprisingly, the absence of TET enzymes and 5hmC did not affect the reactivation of either the IAP-d element or the *Bglap3* gene, suggesting that the DNA methylation status of this retrotransposon does not impact its transcriptional activity (Additional file 3: Figure S3e).

**Fig. 3:**
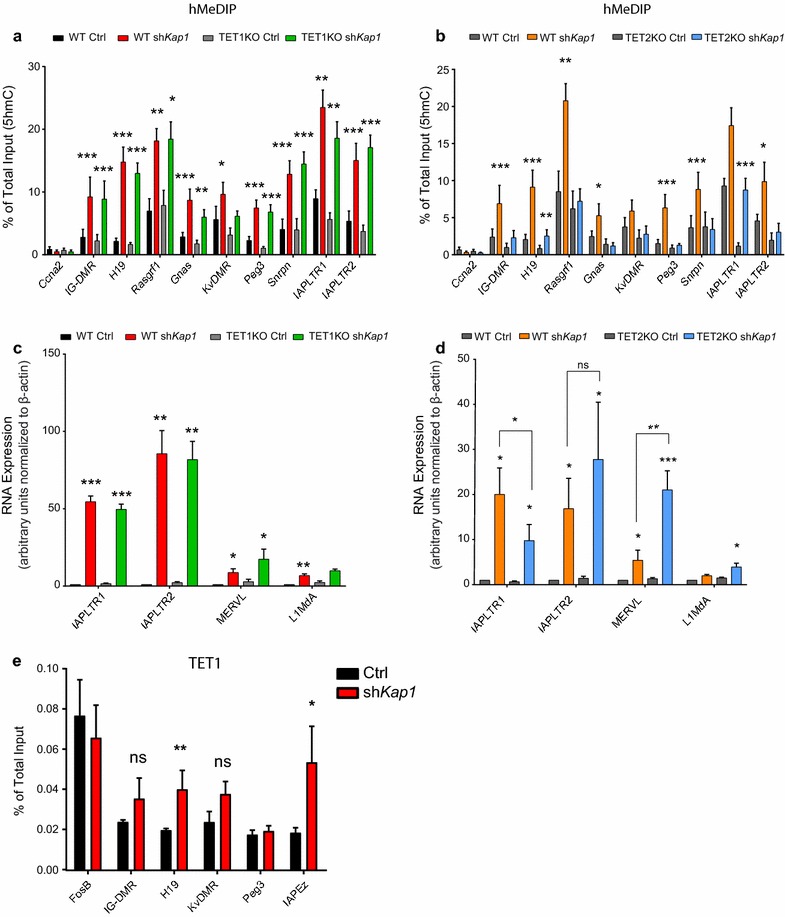
TET2 targets ICRs for 5mC hydroxylation and co-regulates expression of TEs. **a**, **b** 5hmC enrichment measured by hMeDIP-qPCR in TET1 KO cells (**a**), TET2 KO cells (**b**) and their parental cell lines upon *Kap1* knockdown. Error bars represent SEM, *p < 0.05, **p < 0.01, ***p < 0.001, Student’s *t* test. *n* = 4. **c**, **d** Relative expression of indicated TEs measured by RT-qPCR in TET1 KO (**c**) or TET2 KO (**d**) cells and their parental cell lines. Expression was normalized to the housekeeping gene β-actin. Error bars represent SEM, *p < 0.05, **p < 0.01, ***p < 0.001, Student’s *t* test. *n* = 4. **e** ChIP-qPCR for TET1 on mESCs knockdown for *Kap1*. *FosB* promoter is used as a positive control. Error bars represent SEM, *p < 0.05, **p < 0.01, ns = non-significant, Student’s *t* test, *n* = 3

We then sought to identify the TET enzyme responsible for the accumulation of 5hmC at KAP1-bound loci when the master corepressor was depleted. As *Tet3* is not expressed in mESCs [60], we performed *Kap1* knockdown on mESC derivatives deleted for either *Tet1* or *Tet2* [61] (Additional file 4: Figure S4a, b). *Tet1* KO prevented neither the 5hmC-loading nor the transcriptional deregulation of TEs induced by KAP1 removal (Fig. 3a, c). A recent study on primed cells reported binding of TET1 to TEs and ICRs following depletion of SETDB1 and loss of H3K9me3 [62], but here in naïve cells depleted for KAP1 we detected only low levels of TET1 recruitment restricted to the *H19* ICR and to IAPEz (Fig. 3e). In contrast, *Kap1* knockdown in *Tet2* knockout cells abrogated 5hmC acquisition at several ICRs and at IAPEz-LTR2 integrants (Fig. 3b). Interestingly, upregulation of MERVL and IAPEz-LTR2 was greater and that of IAPEz-LTR1 lower upon *Kap1* knockdown in *Tet2* KO than in control cells, suggesting differential role for TET2-mediated regulation on specific subfamilies of retrotransposons (Fig. 3d). A recent report [63] showing TET2-dependent reactivation of IAPs upon SETDB1 removal corroborates our findings. TET2-specific ChIP with antibodies against endogenous TET2 was unsuccessful, and complementation of the KO mESCs with exogenous tagged TET2 protein was lethal, preventing experiments aimed at determining whether KAP1 prevents TET2 genomic recruitment or acts through some other mechanism. However, our data strongly suggest that TET2 is the enzyme responsible for the accumulation of 5hmC at ICRs and numerous TE integrants upon KAP1 depletion in ground-state murine ESCs.

### Accumulation of 5hmC correlates with reactivation of KAP1-controlled TEs

**Fig. 4:**
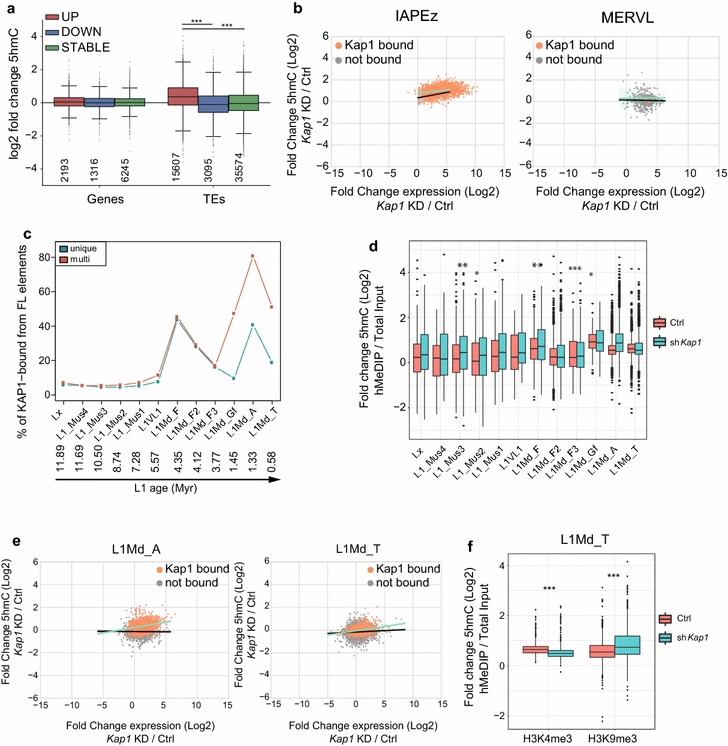
5hmC accumulates at upregulated TEs upon KAP1 removal. **a** Boxplot of 5hmC enrichment fold change between *Kap1* knockdown and control sample on genes or TEs separated in upregulated (brown), downregulated (blue) or stable (green) according to their behaviour upon *Kap1* knockdown as measured by RNA-seq (5 days post-transduction). Number of elements in each subgroup is shown below in each box. Wilcoxon test, ***p < 0.001. Data are averaged from three independent RNA-seq experiments. **b** Correlation plots between fold change in RNA levels and 5hmC enrichment upon *Kap1* knockdown for IAPEz (left) and MERVL (right) integrants. Elements in each family are divided in KAP1-bound (orange) and not bound (grey). Regression line is traced in green for bound elements and black for not-bound elements. IAPEz: bound *p*val = 0.0, Spearman correlation coefficient = 0.3, not bound *p*val = 0.44, Spearman correlation coefficient = 0.2. MERVL: bound *p*val = 0.926, Spearman correlation coefficient = 0.0216, not bound *p*val = 0.4, Spearman correlation coefficient = 0.03. **a** Percentage of KAP1-bound L1 elements from indicated subfamilies in mESCs, arranged from the oldest to the youngest as previously described [65]. KAP1 binding was determined by ChIP-Seq, plotting either uniquely or multiply mapped reads. Data are averaged from two independent ChIP-seq experiments. Myr: Million years. **b** Boxplot of 5hmC enrichment on indicated KAP1-bound L1 elements in wild-type and *Kap1* knockdown cells. Wilcoxon test, *p < 0.05, **p < 0.01, ***p < 0.001. Data are averaged from two independent hMeDIP-seq experiments. **c** Correlation plots between fold change in RNA levels and 5hmC enrichment upon *Kap1* knockdown for L1Md_A and L1Md_T elements. Elements in each family are divided in KAP1-bound (orange) and not bound (grey). Regression line is traced in green for bound elements and black for not-bound elements. L1Md_A: bound *p*val = 0.0, Spearman correlation coefficient 0.26, not bound *p*val = 0.69, Spearman correlation coefficient = 0.01. L1Md_T: bound *p*val = 0, Spearman correlation coefficient = 0.3, not bound *p*val = 0.0, Spearman correlation coefficient = 0.08. **e** Boxplot of 5hmC enrichment on L1MdT KAP1-bound and enriched for the indicated histone mark, in wild-type and *Kap1* knockdown cells. Wilcoxon test, *p < 0.05, **p < 0.01, ***p < 0.001. Data are averaged from two independent hMeDIP-seq experiments

These analyses showed that TET proteins, and in particular TET2, target KAP1-controlled TEs in ESCs, triggering oxidation and loss of 5mC when the master corepressor is absent. To investigate on a global scale whether accumulation of 5hmC correlated with transcriptional reactivation of TEs, we performed deep RNA sequencing of wild-type and TET TKO cells 5 days after *Kap1* knockdown. We performed differential expression analysis and divided genes and TEs in upregulated, downregulated and stable according to their relative expression in *Kap1* knockdown compared to control cells (Fig. 4a). Interestingly, only for the TEs that were upregulated upon *Kap1* knockdown was a significant increase in 5hmC observed, while for genes no significant change was detected, probably due to low levels of methylation over gene bodies at baseline. Moreover, genes are generally devoid of H3K9me3 and enriched for H3K4me3 and H3K27me3 (Additional file 5: Figure S5a), confirming that only KAP1/H3K9me3-regulated sequences are targeted by hydroxymethylation. Members of the highly KAP1-targeted ERVK family displayed a significant correlation between upregulation and increase in 5hmC (Fig. 4b, Additional file 5: Figure S5b). MERVLs, a 2-cell stage-specific family of ERVs [64], were strongly upregulated upon *Kap1* knockdown, although they were neither bound by KAP1 nor enriched for H3K9me3 (Fig. 4b, Additional file 5: Figure S5b). Furthermore, overexpression of these elements, which have a low CpG content, was not accompanied by an increase in 5hmC. Finally, we sought a correlation between hydroxymethylation and reactivation of LINE elements, which we had previously noted to be KAP1-controlled in an age-dependent manner in serum-grown, primed ESC [65]. Here, in ground-state pluripotent stem cells, we found a larger percentage of L1MdA integrants bound by KAP1 than we had previously seen (Fig. 4c), but we still observed that KAP1 enrichment was minimal on the youngest LINE family members. Remarkably, only integrants that were KAP1-bound were upregulated and accumulated 5hmC upon *Kap1* knockdown, in particular for the L1MdA and L1MdF subgroups (Fig. 4d, e, Additional file 5: Figure S5c). We found that about one-third of KAP1-bound L1MdT were enriched for H3K4me3 (Additional file 5: Figure S5d), a mark of active transcription, a feature not shared by more tightly controlled L1s such as L1MdA. When we separated KAP1-bound L1MdT enriched in H3K4me3 or H3K9me3, we observed that only the latter accumulated 5hmC after *Kap1* knockdown (Fig. 4e, f) and that they were also the ones with the strongest increase in expression (Additional file 5: Figure S5e), although their levels of expression remained lower compared to active, non-KAP1-repressed L1MdT. The role of KAP1 binding at actively transcribed, H3K4me3-bearing L1MdT remains unknown.

### KAP1, DNMTs and TETs cooperate to control expression of TEs and imprinted genes

**Fig. 5:**
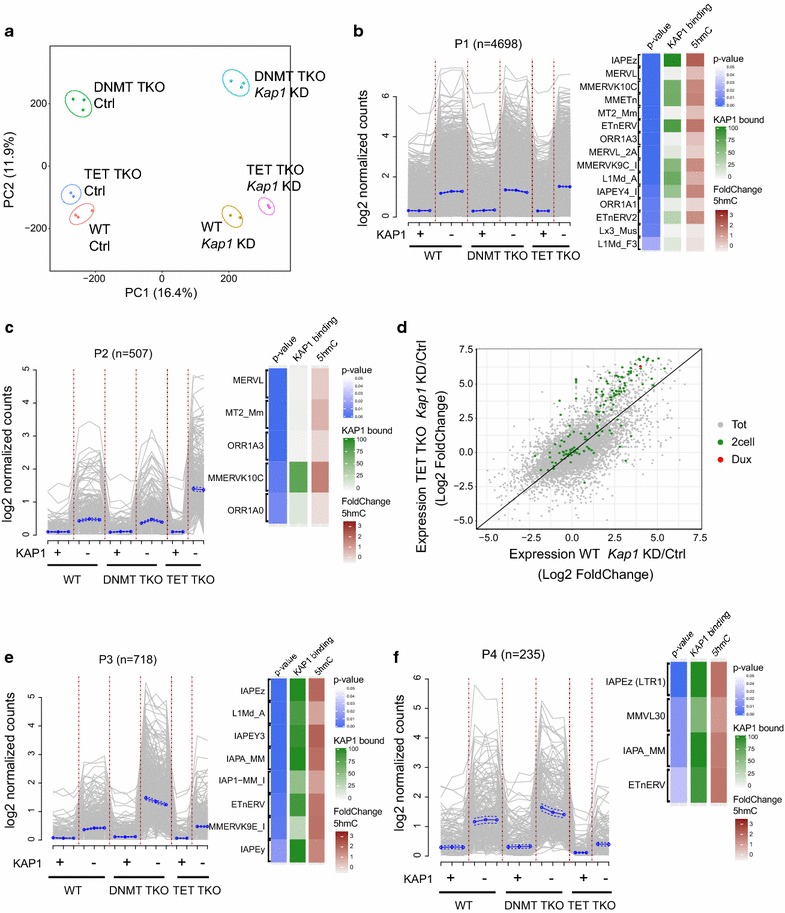
Impact of DNMTs and TET proteins on KAP1-mediated TE regulation. **a** Principal component analysis of TEs expression on data from wild-type, DNMT TKO and TET TKO mESCs control or *Kap1* knockdown, obtained by RNA-seq. Data are from three (for WT and DNMT TKO samples) and two (for TET TKO samples) independent RNA-seq experiments. **b**, **c**, **e** and **f** Graphic representation of logged normalized counts across samples obtained by RNA-seq for TEs grouped in patterns P1 to P4. Families enriched for each pattern are depicted on the right with the *p* value from the hypergeometric test defining how significant over-representation is for each (blue heatmap), the percentage of KAP1-bound elements in the family (green heatmap) and the fold change of 5hmC upon *Kap1* knockdown (red heatmap). **d** Scatter plot of the gene expression fold change between *Kap1* knockdown and control samples for wild-type and TET TKO cells. Mid 2-cell genes are highlighted in green, *Dux* transcript in red

In order to get a more complete view of the relationship between KAP1, DNA methylation and TET-dependent hydroxymethylation in the control of TEs, we compared the transposcriptome, that is the sum of TE-derived transcripts, of either control or *Kap1*-depleted wild-type, *Dnmt* triple knockout (DNMT TKO) and TET TKO cells. Principal component analysis revealed that removal of KAP1 had a major impact on TEs expression in all three genetic backgrounds (Fig. 5a). It also indicated that DNMT TKO cells clustered separately from wild-type and TET TKO cells, suggesting that, after KAP1, DNA methylation plays an important role in controlling TEs. Unsupervised clustering analysis of the 5000 most deregulated TEs revealed that upon *Kap1* knockdown most were upregulated in all three genetic backgrounds (Additional file 6: Figure S6a). We observed a similar behaviour for differentially expressed genes, with DNMT TKO cells clustering separately from WT and TET TKO (Additional file 6: Figure S6b). This suggested that absence of hydroxymethylation has a less dramatic effect on genes and TEs expression than complete absence of DNA methylation. Nonetheless, having noted that TEs accumulate 5hmC upon *Kap1* removal, we explored further the potential implication of TET proteins in TEs regulation. For this, we applied a hierarchical model to categorize without bias the expression of individual TE integrants into patterns of behaviour across the six conditions: wild-type, DNMT or TET TKO cells, either untreated or *Kap1*-depleted. We selected the few patterns contributed by the highest numbers of integrants, and examined their TE composition (Fig. 5b–f). The most common pattern, which we coined P1, was constituted by elements with similar basal levels of expression in wild-type, DNMT and TET TKO cells, and comparable degrees of upregulation following *Kap1* knockdown in all three settings. ERVK family members such as IAPEz, MERVK10C and ETnERV, but also MERVL and L1MdAs, abounded in this P1 subgroup (Fig. 5b). Nevertheless, the expression profile of several MERVL and MaLR (ORR1A) integrants fitted better with pattern P2, where upregulation upon *Kap1* depletion was strongest in TET TKO cells (Fig. 5c). Accordingly, 2-cell (2C) stage genes, the transcription of which is commonly driven by MERVL, were also more upregulated in this setting (Fig. 5d). Interestingly, *Kap1* KD TET TKO cells also expressed higher levels of *Dux*, recently determined to be a KAP1-controlled master activator of the MERVL LTR and of 2C-specific genes, that is, of zygotic genome activation (ZGA) [66, 67] (Fig. 5d). MERVK10C integrants were overrepresented in P2 (Fig. 5c), and both the MERVL and MERVK10C subsets of TEs were also prominent in P5, a subgroup characterized by lower induction upon *Kap1* depletion in DNMT TKO compared to control cells (Additional file 6: Figure S6c). MERVK10C are generally bound by KAP1, although enrichment for the repressor is low compared to strongly controlled elements such as IAPEz and L1MdA (Additional file 6: Figure S6d) and they show a slight increase in 5hmC upon withdrawal of the repressor. Since ERVL elements in patterns P2/P5 seem to be indirectly controlled by KAP1 and TETs, we could speculate for MERVK10C a combination of direct and indirect regulation although the transcriptional activators driving their expression are still unknown. Pattern P3 contained TEs, the repression of which was enforced by both KAP1 and DNA methylation; it harboured many L1MdA elements and ERVs such as IAPEz and IAPEY3, as well as other ERVK family members such as ETnERV and MERVK9E. Other ERVK elements known as tightly controlled by KAP1 such as IAPEz (in particular the ones bearing as LTR an IAPLTR1), ETnERV and VL30 were also enriched in pattern P4, where upregulation following *Kap1* depletion was largely lost in TET TKO cells (Fig. 5f). Some IAPEz elements exhibited an opposite behaviour, being more upregulated in TET TKO cells (pattern P6, Additional file 6: Figure S6e). This subset corresponded to IAPEz elements bearing IAPLTR2 as promoter, and evolutionary older, less active and shorter (2 kb of average size) than their IAPLTR1-driven counterparts [68]. We tried to identify some of the features, such as length and surrounding chromatin marks, which could separate integrants from the same families clustering in different patterns (Additional file 7: Figure S7). For MERVL (Additional file 7: Figure S7a), elements in pattern P2 seem to be slightly shorter and could have accumulated some deletions compared to other integrants, while elements in pattern P5 seem to have integrated farther from genes and H3K27me3-enriched regions. For MERVK10C (Additional file 7: Figure S7b), no striking difference emerged from the analysed features, except for a slightly higher distance between P2 integrants and H3K27me3-enriched regions. In the case of IAPEz (Additional file 7: Figure S7c), we could readily confirm the shorter length of P5 integrants, while P4 integrants seem to be the most associated with highly heterochromatic regions enriched in H3K9me3. For L1MdA (Additional file 7: Figure S7d), longer, full-length elements seem to be more enriched in pattern P3, while truncated integrants abound in pattern P1.

Interestingly, differences in regulation were not limited to TEs but also affected expression of genes. For example, some imprinted genes such as *Meg3* and *Kcnq1* were deregulated upon *Kap1* removal in wild-type cells but not in DNMT TKO cells, consistent with the known DNA-dependent recruitment of KAP1 to these sequences (Additional file 8: Figure S8a, b) [8]. On the contrary, in TET TKO cells, deregulation of these genes was less pronounced, probably due to incomplete erasure of DNA methylation. We also found several differentially expressed genes, the transcription of which was governed by LTRs of upregulated TEs. Expression of genes driven by MERVL-LTRs (such as *Iqcf1* and *Lrrn4*) was higher in TET TKO cells upon *Kap1* knockdown (Additional file 8: Figure S8c–f), while some IAP-LTRs drove stronger expression of nearby genes in DNMT TKO cells (Additional file 8: Figure S8g–i). We confirmed the presence of the corresponding chimeric transcripts, initiated within LTRs and extending into the coding region of these genes, by sequencing of PCR-amplified products (Additional file 8: Figure S8j).

## Discussion

Our study sheds new light on the modalities of KZFP/KAP1-mediated heterochromatin maintenance at TEs and ICRs in ground-state murine pluripotent stem cells. We first found that regions bound by KAP1 and enriched in H3K9me3 retained high levels of DNA methylation in the otherwise generally demethylated genomic landscape of naïve mouse ES cells, and that removal of KAP1 resulted in loss of H3K9me3 and decreases in CpG methylation at these sites. This is consistent with the previous demonstration that (1) docking of the KRAB/KAP1 complex to genomic loci during the first few days of mouse embryogenesis leads to methylation of the underlying DNA [69]; (2) KAP1 can associate with both de novo and maintenance DNA methyltransferases and with the hemimethylated DNA-binding protein UHRF1/NP95 [8] and ZFP57 requires its cofactor to recruit DNA methyltransferases and maintain DNA methylation imprint in embryonic stem cells via its transcriptional repression domain [70]; (3) KAP1-binding sequences, for instance derived from ERVs, can trigger CpG methylation on a nearby promoter in mESCs [71, 72], and (4) H3K9me3-bearing regions are resistant to DNA demethylation during conversion of mESCs from serum to 2i condition in an NP95/UHRF1-dependent fashion [54]. Together, these evidences support a model whereby loss of a KZFP-tethered, KAP1-nucleated heterochromatin- and DNA methylation-inducing/maintaining complex results in passive loss of DNA methylation at ICRs and TEs. Our data further reveal that, upon *Kap1* depletion, 5hmC accumulates at loci normally enriched for the corepressor and H3K9me3, including ICRs and numerous TEs. These genomic targets similarly acquire 5hmC when their cognate KAP1-docking KZFP is deleted, namely ZFP57 for ICRs and ZFP932 for ERVK integrants. Accumulation of 5hmC was restricted to methylated DNA loci, as expected from the need for its 5mC precursor. It was previously noted that 5hmC is gained at some ICRs and TEs in G9a- or SETDB1-depleted murine ES cells [62, 73], and it was suggested that part of this effect might stem from the normally protective effect of the H3K9me2-binding PGC7 protein (also known as Stella or DPPA3) against the chromatin recruitment of TET [73].

The KZFP/KAP1 system is essential to preserve imprinting from erasure and to control the transcriptional activity of transposable elements during the wave of genomic reprogramming that takes place during early embryogenesis. Maternal-zygotic *Kap1* knockout results in highly penetrant early embryonic lethality, whereas its maternal-only counterpart is characterized by variable ICR methylation defects, an attenuation of phenotype likely resulting from *Kap1* expression from the paternal allele following zygotic genome activation [18, 74]. Maternal and zygotic knockout of *Zfp57*, the KZFP responsible for recruiting KAP1 at methylated ICRs, leads to loss of imprinting at several but not all of these loci [7], while stable *Zfp57* knockout mESCs display complete loss of KAP1 binding, H3K9me3 enrichment and DNA methylation at all germ line ICRs [8]. Here, we found that upon *Zfp57* knockdown several ICRs rapidly accumulated 5hmC and underwent DNA demethylation. Loss of DNA methylation occurred both in the presence and absence of TET proteins, but was less pronounced in the latter setting, suggesting the contribution of both passive and active demethylation to this process.

TET1 and TET2 are the main TET enzymes expressed in blastocyst and mESCs, where others and we have detected little if any TET3 [60]. A recent study reported that TET1 binds to and demethylates ICRs and TEs upon loss of H3K9me3 induced by knockout of SETDB1 in primed ES [62], but we found here that levels of 5hmC loading at ICRs and TEs as well as TEs transcriptional activation upon *Kap1* knockdown were similar in wild-type and *Tet1* knockout cells. Moreover, we did not find significant TET1 recruitment at KAP1-deprived loci. This could be linked to the different culture condition, as a recent study reported differences in the role of TET1 and TET2 between primed and naïve cells [63]. In contrast, *Tet2* knockout mES cells depleted for KAP1 failed to accumulate 5hmC at ICRs and TEs, and displayed patters of TE deregulation comparable to those recorded in TET TKO cells, with higher levels of expression of MERVLs and reduced IAPEz-LTR1 activation. We conclude that TET2 is the main 5mC oxidase countered by KAP1 in ground-state murine ES cells.

While DNA methylation at ICRs is necessary to regulate parent-of-origin-restricted expression of imprinted genes, its role in the epigenetic regulation of transposable elements is not as clear-cut. It had long been assumed that DNA methylation is established at TEs during early development and subsequently maintained throughout differentiation to ensure their irreversible silencing in adult tissues. Recent studies have invalidated this model, even providing evidence that retrotransposons can be reactivated without losing DNA methylation [28–30, 75]. Complete abrogation of this modification in murine ESCs by knockout of both de novo and maintenance DNA methyltransferases (DNMT TKO) results in only mild upregulation of a remarkably small subset of integrants [44]. Acute depletion of these DNMTs leads to induction of some IAP and L1 elements, but they are rapidly repressed by reorganization of repressive histone marks [76, 77]. The present work adds to these data by unveiling an unsuspected level of complexity in the relative contribution of KZFP/KAP1-induced repressive chromatin marks, DNA methylation and TET-mediated 5mC oxidation to the transcriptional control of TEs. Active demethylation can relieve repression by removing 5mC, but 5hmC could in itself activate expression by allowing the recruitment of chromatin modifiers and transcription factors [78, 79]. Conversely, TET proteins were recently proposed to repress young L1s by attracting SIN3A and the NuRD complex [80] or to regulate IAPs expression by indirectly controlling deposition of H4R3me2 [63]. Through a comparison of wild-type, DNMT and TET TKO murine ES cells, we found that *Kap1* depletion activated TEs in all three settings, but with important nuances. For many TEs (pattern P1), KAP1 appeared as the main repressor, with little contribution of DNA methylation. For some integrants (pattern P2), TET proteins reinforced KAP1 action, as reflected by greater induction upon *Kap1* knockdown in TET TKO than in control cells. These TEs included class III ERVs such as MERVLs and ORR1As, the LTR of which is responsible for driving the expression of 2-cell stage genes during ZGA yet does not recruit KAP1. Explaining this phenomenon, we found that induction of *Dux*, the recently identified KAP1-repressed master regulator of ZGA [66, 67], was amplified in TET TKO cells. An ERVK family member, MERVK10C, also exhibited higher expression in the absence of TET proteins and little responsiveness to lack of DNA methylation. MERVK10C integrants are amongst the most commonly upregulated retroelements following *Kap1* depletion in differentiated tissues, generally without changes in their DNA methylation [28, 29]. These ERVs are not strongly enriched in KAP1, and their activation upon KAP1 withdrawal is likely dependent on the chromatin environment of specific integrants or on indirect effects on their transcriptional activators, most of which are yet to be identified. A third group of TEs were more upregulated upon *Kap1*-depletion in the absence of DNA methylation (pattern P3), for instance L1MdA and other ERVK family members such as IAPEz, ETnERV and IAPEY. These ERVKs were conversely less upregulated in TET TKO cells, further supporting a role for DNA methylation in their control. Finally, some TEs were less activated upon *Kap1* depletion in TET TKO than in control or DNMT TKO cells (pattern P4). It suggests that, for these elements, TET proteins, 5hmC or secondary products of active demethylation might play a positive role in the recruitment of transcriptional activators. Noteworthy, amongst IAPEz, integrants with different LTRs displayed distinctive behaviours. DNA methylation played a more significant role in the control of the younger and generally more active IAPLTR1-containing IAPEz, the *Kap1* depletion-induced activation of which was also, for some, reduced in the absence of TET proteins (pattern P4). In contrast, loss of 5mC oxidation did not affect the susceptibility of older IAPLTR2-containing IAPEz or L1MdA elements to *Kap1* removal (pattern P6 and P3 and Additional file 6: Figure S6d).

## Conclusions

In sum, the present work demonstrates that the KZFP/KAP1 system plays a critical role in preserving histone and DNA methylation at ICRs and TEs in naïve embryonic stem cells. Our study also reveals that the respective contribution of KZFP/KAP1-driven chromatin modifications, TET-mediated 5mC hydroxylation and DNA methylation to the control of endogenous retroelements can greatly vary, warranting that, when studying the regulation of TEs, consideration should be given to the sequence of individual integrants, to their genomic location and to the set of transcriptional repressors and activators recognizing their provirus, rather than to their belonging to one or another general class of transposons.

## Methods

### Cell culture

All murine embryonic stem cells were cultured in 2i + LIF media as previously described [81]. Cells knockout for *Dnmt1*, *Dnmt3a*, *Dnmt3b* [82] were obtained from the group of Professor Masaki Okano. Cells knockout for *Tet1*, *Tet2* and *Tet3* [83] and single knockout for *Tet1* and *Tet2* along with their parental cell lines [61] were obtained from the group of Professor R. Jaenisch. Cast/129 hybrid mESCs were kindly provided by the group of Todd Macfarlan. Cells were transduced, selected with 4 μg/mL puromycin and collected after 5 days.

### Plasmids and lentiviral vectors

pLKO.puro shRNA vectors were used for KAP1 and ZFPs KD. The shRNA for *Zfp932/Gm15446* and *Kap1* were previously described [28] The shRNA for *Zfp57* was obtained from the RNAi Consortium (http://www.broadinstitute.org/rnai/public/). All shRNAs sequences are listed in Additional file 9: Table S1. Lentiviral vectors production protocols are detailed at http://tronolab.epfl.ch, and backbones are available at Addgene (http://www.addgene.org).

### ChIP-PCR and ChIP-seq

Cells were harvested, washed with Episerf (LifeTechnologies, #10732), fixed in 10 mL per 1 × 107 cells (10 min in 1% formaldehyde), quenched with TrisHCl in 50 mL (at 250 mM final), washed with PBS and pelleted. For TET1 ChIP, cells were fixed with an initial cross-linking step of 45 min with mM Di(N-succinimidyl)glutarate (Sigma-Aldrich, #80424) in PBS at room temperature followed by formaldehyde fixation, as previously described [80]. Each pellet containing 1 × 107 cells was lysed, resuspended in 1 mL of sonication buffer on ice (10 mM Tris at pH 8, 200 mM NaCl, 1 mM EDTA, 0.5 mM EGTA, 0.1% NaDOC, 0.25% NLS, and protease inhibitors), transferred to TC 12 × 12 tubes (Covaris) and sonicated (Covaris settings 20 min, 5% duty cycle, 140 W, 200 cycles). Sonication was assessed by reverse cross-linking (65 °C, RNAse A at 1 μg/μL, overnight), followed by DNA extraction. Fragment size (between 200 and 400 bp) was checked on a Bioanalyzer (Agilent 2100). Immunoprecipitations were performed with chromatin from 1 × 107 cells with Dynabeads (ThermoFisher) in IP buffer (16.25 mM Tris at pH 8.1, 137.5 mM NaCl, 1 mM EDTA, 0.5 mM EGTA, 1.25% Triton X-100, and protease inhibitors) overnight. Chromatin was reversed cross-linked (65 °C, proteinase K at 400 ng/μL, overnight), and DNA was further extracted for analysis. Antibodies used were H3K4me3 (Cell Signalling Technology, #9751), H3K27me3 (Abcam, #6002) and TET1 (GeneTex, #GTX125888). ChIP samples were used for SYBER Green qPCR (Applied Biosystems) or library preparation for sequencing. Two replicates from independent experiments for each sample were used for high-throughput sequencing. Primers were designed using Primer 3 [84]. All primers sequences are listed in Additional file 9: Table S1. Libraries of immunoprecipitated chromatin and total input control from ChIP were performed with paired-end adaptors as previously described [28]. Sequencing was performed on an Illumina NextSeq 500 (Illumina), with each library sequenced in 75-bp reads paired-end run.

### DNA methylation analysis

Genomic DNA was extracted, converted using an Epitect Bisulfite kit (Qiagen) and used in two rounds of PCR followed by PCR product purification. Bisulfite sequencing was performed as previously described [28]. Quantification of methylation was performed with QUMA [85]. glucMS-qPCR assay was performed using the EpiJET 5-hmC and 5-mC Analysis Kit (ThermoFisher, #K1501) according to manufacturer instructions. For Additional file 2: Figure S2a, b, glucMS-qPCR was performed on KAP1-immunoprecipitated DNA or input DNA after sonication. MeDIP and hMeDIP were performed as previously described [86]. Genomic DNA was extracted by lysing the cells (20 mM Tris–HCl pH 8.0, 4 mM EDTA, 20 mM NaCl, 1% SDS), purification of nucleic acids by phenol–chloroform extraction followed by precipitation with Na acetate, resuspension in TrisEDTA and RNase A treatment. DNA was sonicated either with Branson sonifier (5 × 10 s amplitude 20%) or Covaris (Peak Incident power 140, Duty factor 10%, cycles per burst 200, treatment time 100 s) to obtain 200–500-bp fragments. Sonicated DNA was then denatured and immunoprecipitated with antibodies against 5mC (Diagenode, #Mab-006) or 5hmC (Active Motif, #39769) overnight at 4 °C in IP buffer (100 nM Na-Phosphate buffer pH 7.0, 1.4 M NaCl, 0.5% Triton X-100). DNA was purified and used for SYBER Green qPCR (Applied Biosystems). Primers for bisulfite sequencing were designed using MethPrimer [87]. All primers sequences are listed in Additional file 9: Table S1. Two replicates from independent experiments for each sample were used for high-throughput sequencing. For high-throughput sequencing, the steps of end repairing, A-tailing and adapter ligation were performed before denaturation and immunoprecipitation, while size selection and library amplification were performed after. Libraries of immunoprecipitated chromatin and total input control from hMeDIP were performed with single-end adaptors as previously described [28]. Sequencing was performed on an Illumina HiSeq 2500 (Illumina), with each library sequenced in 100-bp reads run.

### RT-qPCR and RNA-seq

Total RNA was extracted and DNAse-I treated using a spin column-based RNA purification kit (Macherey–Nagel). Reverse transcription was performed with 500 ng of RNA using random primers and SuperScriptII (Invitrogen). Primers were designed using Primer 3 [84] and used for SYBER Green qPCR (Applied Biosystems). All primers sequences are listed in Additional file 9: Table S1. For mRNA sequencing, 100-bp RNA-seq libraries were prepared using 200 ng of total RNA and the Illumina TruSeq Stranded mRNA reagents (Illumina). Three replicates from independent experiments for each sample for wild-type and DNMT TKO cells and two replicates for TET TKO cells were selected for high-throughput sequencing. Cluster generation was performed with the resulting libraries using the Illumina TruSeq SR Cluster Kit v4 reagents and sequenced on an Illumina HiSeq 2500 (Illumina).

### Immunoblotting

Cells were washed with ice-cold PBS and resuspended in radioimmunoprecipitation (RIPA) buffer to prepare total cell extracts. Protein amount was quantified by BCA protein assay reagents (Pierce) and normalized for loading on a 10% denaturing SDS–polyacrylamide gel. Wet transfer was performed, and the primary antibodies used were anti-Trim28 (mouse mAb; MAB3662 Millipore), anti-ZFP57 (ab45341, Abcam) and anti-beta Actin HRP (ab20272, Abcam).

### Allelic discrimination

Genomic DNA or DNA from hMeDIP on hybrid Cast/129 (Cast/EiJ male × 129/Sv female) murine embryonic stem cells was subjected to PCR (25 cycles, Tm 58°) with primers recognizing both strains. Obtained bands were run on agarose gels, purified and sent for Sanger sequencing to identify SNPs.

### Bioinformatic and statistical methods

All genome-wide TE analyses were performed using a merged repeats track generated in house (by using RepeatMasker 3.2.8 and merging homonymous ERV-int integrants with attributed LTRs within 400 bp or less, for details see Ref. [28]. Genomic coordinates for ICRs have been taken from Strogantsev et al. [88] and are listed in Additional file 10: Table S2. Genomic region analyses were performed with BEDTools [89]. For Fig. 1d, a 25% reciprocal overlap was required between KAP1 and its target. Otherwise, the 1-bp overlap default of BEDTools was used. R version 3.1.2 (http://www.R-project.org) or GraphPad Prism version 4.0 (http://www.graphpad.com) was used for statistical analyses.

#### RNA-seq

RNA-seq reads were aligned to the mm9 genome assembly using HISAT2 [90] with parameters -rna-strandness R. Reads that were not uniquely mapped were discarded from the analysis using bamtools filter v2.4.1 with parameters -tag “NH:1”. Read summarization on genes was generated using featureCounts [91] with parameters -s 2 -t exon -g gene_id -Q 10. TE counts were computed using the multicov script from the BEDTools software with the -split and -s option. Only genes or TEs that had at least as many reads as samples present in the analysis were considered further. Sequencing depth normalization and differential expression analyses were performed using the voom function from the R package LIMMA from Bioconductor [92]. The gene library sizes as given by voom were used to normalize the TEs counts. *p* values were computed using a moderated *t* test and corrected for multiple testing using the Benjamini–Hochberg method [93]. To be considered significantly upregulated, a gene or a TE had to have twofold increased expression and an adjusted *p* value lower than 0.05.

#### ChIP-seq analyses

For previously published datasets, raw data are available at GSE94323 (KAP1 and H3K9me3) and GSM1916143/GSM1916143 (ZFP932). Reads were mapped to the mouse genome assembly mm9 using Bowtie2 short read aligner [94], using the—sensitive-local mode. The peaks were called using either the MACS program v1.4.2.1 [95] or the SICER software v1.1 for the histone modification mark H3K9me3 [96], with the total input chromatin coverage as control. For MACS, we used the default software parameters and selected MACS score above 50. For SICER, we used the recommended parameters for histone marks (redundancy threshold 1, window size 200, fragment size 150, effective genome fraction 0.74, gap size 400, FDR 0.05). KAP1 genomic coverage (0.9% of the genome) was obtained with the *genomecov* command of the bedtools2 suite. For KAP1 enrichment heatmaps (Additional file 6: Figure S6d), we used deeptools2 [97] for the computation of the matrix from ChIP-seq signals and plotting of the heatmap. For the schematic representation of chimeric transcripts in Additional file 8: Figure S8, BAM files from RNA-seq data were loaded on IGV software [98, 99] and tracks for normalized coverage and splicing junctions were shown. For Additional file 7: Figure S7, the distance to the next feature was computed using BEDTools and plotting was performed in R.

#### Hydroxy-MeDIP

The same mapping procedure as for ChIP-seq analysis was used. Bedtools multicov was used for quantifying reads on contiguous regions of interest, and HTSeq-counts were used for quantification on genes. Sequencing depth normalization and fold changes over the inputs were computed using the voom function of the LIMMA package similarly to the RNA-seq analysis. Boxplots and correlation plots were done in python with the matplotlib library. For boxplots, *p* values were calculated using a two-sided student’s *t* test. If the sample size differed by more than 1000, cross-validation was used (number of cv 10,000; subset size 1000) to avoid sample size bias. For the correlation plots, statistical significance was inferred with Spearman correlation.

#### Reduced-representation bisulfite sequencing

Processed data were acquired from the gene expression omnibus entry GSM2051573 and GSM2051574. For each category of interest, the methylation status of each CpG of each feature belonging to the category was extracted. For each feature, the third quartile (q75) of percentage of methylation was computed across the CpGs. Finally, a violin plot representing the distribution of the quartile for each feature in each category was plotted using the seaborn library of python.

#### Clustering analysis

The GaGa R library [100] was used for the expression pattern clustering. In short, GaGa implements a Gamma–Gamma hierarchical model which is fitted to the expression data. After the fit, all possible expression pattern combinations are computed and each feature is assigned to the pattern it is most likely to belong to. Prior to running GaGa, the TEs with a low coverage of less than 20 reads were discarded. Raw data and family enrichment data for all patterns generated are provided as supplementary information in Additional file 11.

#### Coverage plots

ChIP-seq signals were converted to bigwig and normalized to reads per 100 million mapped reads, and then signals on each feature of interest were extracted using the pyBigWig library of python. After resampling to achieve same signals length, the average and a 95% confidence interval around the mean were computed and plotted using the matplotlib library of python.

#### Heatmaps

Heatmaps were generated from the normalized RNA-seq data with the R function heatmap.2. Both rows and columns were clustered using hierarchical clustering with agglomeration method “complete” and distance metric set as Pearson distance.

## Additional files

**Additional file 1: Figure S1.** KAP1 removal leads to accumulation of 5hmC at ICRs and TEs.

**Additional file 2: Figure S2.** KAP1-bound chromatin is protected from 5mC hydroxylation.

**Additional file 3: Figure S3.** Depletion of ZFP57 and ZFP932 results in loss of imprinting and deregulation of TEs and nearby genes.

**Additional file 4: Figure S4.** Kap1 knockdown in* Tet1* and* Tet2* knockout cells.

**Additional file 5: Figure S5.** Correlation between loss of H3K9me3, accumulation of 5hmC and transcriptional deregulation on genes and TEs.

**Additional file 6: Figure S6.** Impact of DNMTs and TETs on KAP1-regulated TEs and genes.

**Additional file 7: Figure S7.** Analysis of genomic features on TE-integrants of the same families clustering in different patterns.

**Additional file 8: Figure S8.** Deregulation of imprinted genes and TE-regulated genes upon Kap1 knockdown in absence of DNA methylation or TET proteins.

**Additional file 9: Table S1.** List of primer sequences.

**Additional file 10: Table S2.** Genomic coordinates of ICRs used in this study.

**Additional file 11.** Pattern analysis.

## Abbreviations

5hmC: 5-hydroxymethylcytosine
5mC: 5-methylcytosine
ChIP: chromatin immunoprecipitation
DNMT: DNA methyltransferase
ERV: endogenous retrovirus
H3K27me3: tri-methylation of Lysine 27 on histone H3
H3K4me3: tri-methylation of Lysine 4 on histone H3
H3K9: histone 3 Lysine 9
H3K9me3: tri-methylation of Lysine 9 on histone H3
(h)MeDIP: (Hydroxy)-methylated DNA immunoprecipitation
IAP: intracisternal A-particles
ICR: imprinting control region
KAP1: KRAB-associated protein 1
KRAB-ZFP/KZFP: Krüppel associated box zinc finger protein
LINE: long interspersed element
LTR: long-terminal repeat
NuRD: nucleosome remodelling and deacetylation complex
OGT: *O*-linked *β*-d-*N*-acetylglucosamine transferase
PRC2: Polycomb repressive complex 2
SETDB1: SET domain bifurcated 1
SINE: short interspersed element
siRNA: small-interfering RNA
SVA: SINE-R/VNTR/Alu
TDG: thymine DNA glycosylase
TE: transposable element
TET: ten-eleven translocation
UHRF1: ubiquitin like with PHD and ring finger domains 1
