# Supplementary material for: Individual retrotransposon integrants are differentially controlled by KZFP/KAP1-dependent histone methylation, DNA methylation and TET-mediated hydroxymethylation in naïve embryonic stem cells
Source: Epigenetics Chromatin. 2018 Feb 26;11:7. doi: 10.1186/s13072-018-0177-1 (PMC6389204; doi:10.1186/s13072-018-0177-1)
Supplement: Supplementary file 6 — Additional file 6: Figure S6. Impact of DNMTs and TETs on KAP1-regulated TEs and genes. [file 13072_2018_177_MOESM6_ESM.pdf]

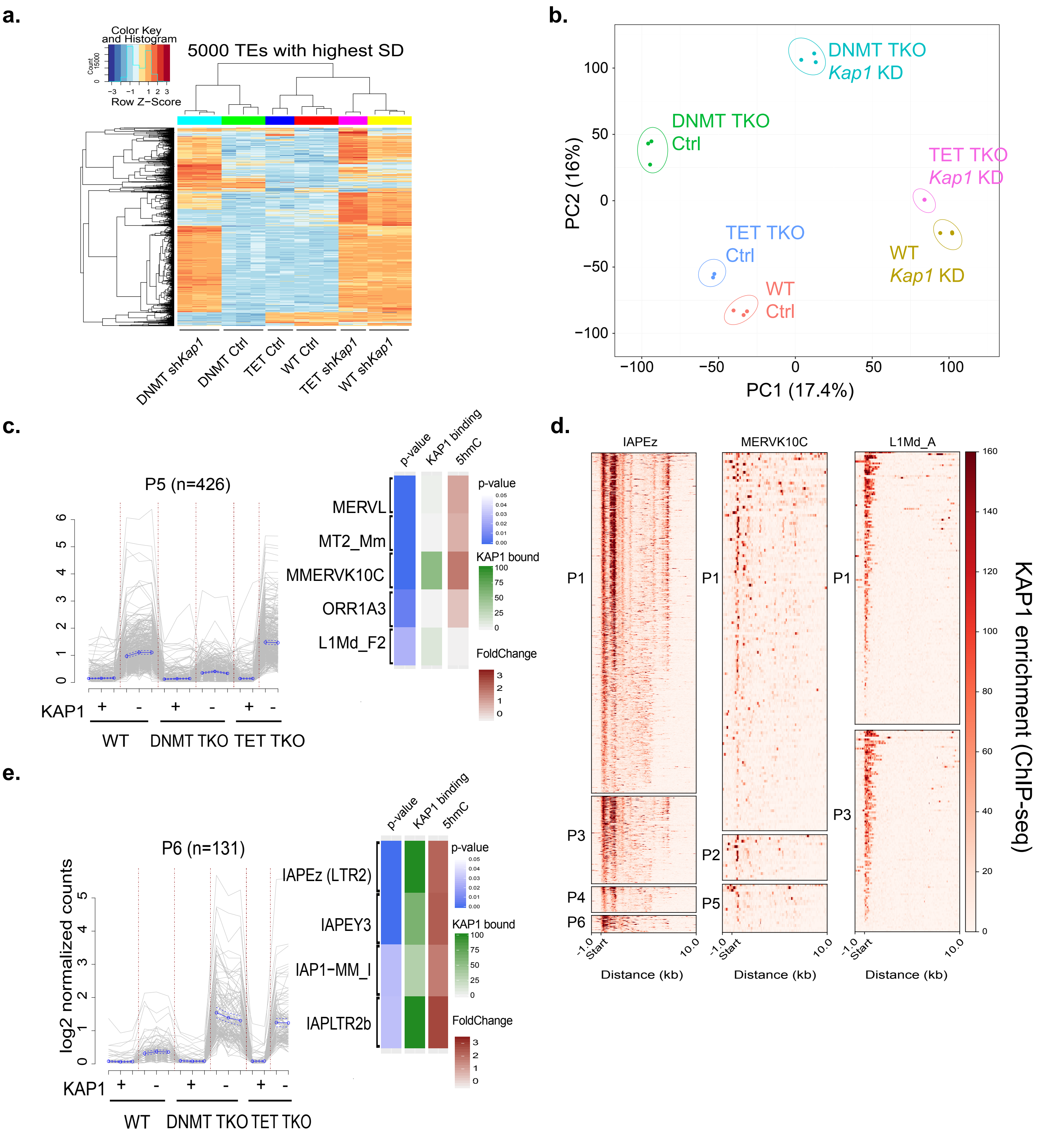

Figure S6. Impact of DNMTs and TETs on KAP1-regulated TEs and genes. a) Expression heatmap of the 5,000 TEs with highest standard deviation. DNMT: DNMT TKO; TET: TET TKO. Color key is indicated on top right, blue downregulation, red upregulation Data are from 3 (WT and DNMT TKO) or 2 (TET TKO) independent RNA-seq experiments. b) Principal component analysis of genes expression on data from wild type, DNMT TKO and TET TKO mESCs control or *Kap1* knockdown, obtained by RNA-seq. Data are from 3 (for WT and DNMT TKO samples) and 2 (for TET TKO samples) independent RNA-seq experiments. c,e) Graphic representation of logged normalized counts across samples for TEs grouped in patterns P5 (c) and P6 (e). Families enriched for each pattern are depicted on the right with the *p* value from the hypergeometric test defining how significant over-representation is for each (blue heatmap), the percentage of KAP1-bound elements in the family (green heatmap) and the fold change of 5hmC upon *Kap1* knockdown (red heatmap). d) Heatmaps showing KAP1 enrichment signal (ChIP-seq) over IAPEz, MERVK10C and L1MdA (1kb upstream and 10kb downstream the beginning of the element). Integrants were separated by pattern of transcirptional behaviour. Data are representative of 2 independent ChIP-seq experiments.
