# Supplementary material for: Individual retrotransposon integrants are differentially controlled by KZFP/KAP1-dependent histone methylation, DNA methylation and TET-mediated hydroxymethylation in naïve embryonic stem cells
Source: Epigenetics Chromatin. 2018 Feb 26;11:7. doi: 10.1186/s13072-018-0177-1 (PMC6389204; doi:10.1186/s13072-018-0177-1)
Supplement: Supplementary file 9 — Additional file 9: Table S1. List of primer sequences. [file 13072_2018_177_MOESM9_ESM.docx]

**Table S1.** List of primer sequences.

| **shRNAs** |  |
| --- | --- |
| shKAP1 | CCGGCCGCATGTTCAAACAGTTCAACTCGAGTTGAACTGTTTGAACATGCGGTTTTTG |
| shZfp932 | CCGGCCTCTCATGGTCAACTTCAAACTCGAGTTTGAAGTTGACCATGAGAGGTTTTTG |
| shZfp57 | CCGGCCAACACACTCAGAATGCAAACTCGAGTTTGCATTCTGAGTGTGTTGGTTTTTG |
| **glucMS-qPCR** |  |
| FosB F | GAG ATT GAC AGG CGC CTA AA |
| FosB R | GGT CCC AGG GGT ATA AGC AG |
| IG-DMR F | AATGGGATCACGCGAGTAAG |
| IG-DMR R | TCCATCAGGAATTCCAAAGC |
| H19 F | CCACATGCACTGGTTTATGG |
| H19 R | GGGTTCACCTGTTTTGCACT |
| Gnas F | ACCCCAAAGGCAAACAATTA |
| Gnas R | GCTCAGAGCTGCAGGAGTTT |
| KvDMR F | ACGTGGACGCAAAATACGAG |
| KvDMR R | CAGTGGCTCCGTATTCGTTT |
| Peg3 F | TAGCCTCTCCCTCCAGACAA |
| Peg3 R | GGGTCTTTTGACCCTGGACT |
| IAPEz F | CTCGCTCGCTGCCTAAAG |
| IAPez R | AAACGAAAGCCCCCTTCTTA |
| **ChIP-qPCR** |  |
| FosB F | GAG ATT GAC AGG CGC CTA AA |
| FosB R | GGT CCC AGG GGT ATA AGC AG |
| IG-DMR F | TGTACACAATGCTGCCGTTC |
| IG-DMR R | CTCGCTAGTTCACGGAGGTC |
| H19 F | GCA CAG CGT GGA GAG TGA AC |
| H19 R | CAT TTC TTG GGT AGC TCC TTC AG |
| KvDMR F | AAACGAATACGGAGCCACTG |
| KvDMR R | GCGGGTTTCTTCTCTGAGTC |
| Peg3 F | GCC ACT GCG GCA AAA CA |
| Peg3 R | GGT CTT CGC AAT CTA GCC ATC T |
| IAPEz F | ACGGGAACACTTCATTACCACC |
| IAPEz R | TTGAGAAGGATTCAACTGCGTG |
| **RT-qPCR** |  |
| Actin F (ActinB) | CTAAGGCCAACCGTGAAAAGAT |
| Actin R (ActinB) | CACAGCCTGGATGGCTACGT |
| TBP F | TTGACCTAAAGACCATTGCACTTC |
| TBP R | TTCTCATGATGACTGCAGCAAA |
| KAP1 F | CGGAAATGTGAGCGTGTTCTC |
| KAP1 R | CGGTAGCCAGCTGATGCAA |
| Zfp57 F | AGT GAT CCA GGG ACC AGT GG |
| Zfp57 R | TGA CAT TCA GGG TCG AGG CT |
| Bglap3 F | CTGACAAAGCCTTCATGTCC |
| Bglap3 R | TCAAGCTCACATAGCTCCC |
| IAP Bglap F | AGGTGTTGCAGAGGTTTTGG |
| IAP Bglap R | AATATCGGACACAGGGCAAG |
| ZFP932/Gm15446 F | TTGCACATCATTGTCATCTCC |
| ZFP932/Gm15446 R | CTGACCTACAAAGGCTTTACCAC |
| Nanog F | GCA AGC GGT GGC AGA AAA |
| Nanog R | GGT GCT GAG CCC TTC TGA ATC |
| Tet1 F | TCT CTC ATA GAG TGA GCT AGC ACG TAA |
| Tet1 R | GCC CGC TGA GTC CTG TAA AC |
| Tet2 F | CCGTCGCTGTTCTCAGAATG |
| Tet2 R | GTCTCTCTTCCTCTTTTGGCTCA |
| **(h)MeDIP** |  |
| Ccna2 F | AGT AGC CCG CGA CTA TTG AAA T |
| Ccna2 R | GCG ACC GGC GCT TCT |
| IG-DMR F | TGTACACAATGCTGCCGTTC |
| IG-DMR R | CTCGCTAGTTCACGGAGGTC |
| H19 F | GCA CAG CGT GGA GAG TGA AC |
| H19 R | CAT TTC TTG GGT AGC TCC TTC AG |
| Rasgrf1 F | CTG CTG CTC CCA CAT CCA T |
| Rasgrf1 R | GCA GTC GTG GTA GTT GTA GC |
| KvDMR F | AAACGAATACGGAGCCACTG |
| KvDMR R | GCGGGTTTCTTCTCTGAGTC |
| Snrpn F | CTG ACC TTC CTC GCT CCA TT |
| Snrpn R | GAC TAG CGC AGA GAG GAG AG |
| Gnas F | TGC CCA GGA ATA ATC TGC AGA |
| Gnas R | ATA CAG TCA CAT TGC CCG GT |
| Peg3 F | TAGCCTCTCCCTCCAGACAA |
| Peg3 R | GGGTCTTTTGACCCTGGACT |
| Grb10 F | CAT ACG TGT TAC ATG CGC CA |
| Grb10 R | TGT CGG TTC GTT TAG GAG CT |
| IAPEz F | ACGGGAACACTTCATTACCACC |
| IAPEz R | TTGAGAAGGATTCAACTGCGTG |
| BglapProm F | TCATGGTGTCTGCTAGGTGTG |
| BglapProm R | TCAGAATCAGAGGCAACAGG |
| IAP Bglap Kap1peak F | AGGTGTTGCAGAGGTTTTGG |
| IAP Bglap Kap1peak R | AATATCGGACACAGGGCAAG |
| IAP Bglap 5'LTR F | TTGGTGCACTGTTTGACCTG |
| IAP Bglap 5'LTR R | AATAAGGTTCCCGGTCTTGG |
| **Chimeric transcripts** |  |
| Ugt3a2 F | ACTGCGGGACAAATTACAGC |
| Ugt3a2 R1 | ACTTGCGACACACGGCTTAT |
| Ugt3a2 R2 | ACACAAATCGTTTCCCAGC |
| 17000A1804Rik F1 | TGAATTGCCTGCTGTGGTAG |
| 17000A1804Rik F2 | AACCTCGTTCCTGGTCCTTT |
| 17000A1804Rik R | CTGGGGAACGAGAGTACCAG |
