# Supplementary material for: Individual retrotransposon integrants are differentially controlled by KZFP/KAP1-dependent histone methylation, DNA methylation and TET-mediated hydroxymethylation in naïve embryonic stem cells
Source: Epigenetics Chromatin. 2018 Feb 26;11:7. doi: 10.1186/s13072-018-0177-1 (PMC6389204; doi:10.1186/s13072-018-0177-1)
Supplement: Supplementary file 10 — Additional file 10: Table S2. Genomic coordinates of ICRs used in this study. [file 13072_2018_177_MOESM10_ESM.docx]

**Table S2.** Genomic coordinates of ICRs used in this study are taken from Strogantsev et al., 2015.

| **chromosome** | **start** | **end** | **ICR name** | **KAP1 binding** |
| --- | --- | --- | --- | --- |
| chr17 | 12934169 | 12935816 | Igf2r | + |
| chr6 | 30684932 | 30689966 | Mest | + |
| chr11 | 22871610 | 22874212 | Zrsr1/Commd1 | + |
| chr12 | 110762703 | 110773093 | IGDMR | + |
| chr2 | 157385609 | 157387535 | Nnat/Peg5 | + |
| chr10 | 12809697 | 12812131 | Zac1/Plagl1 | + |
| chr2 | 174119863 | 174126564 | GnasProXL | + |
| chr6 | 58856396 | 58857391 | Nap1l5 | + |
| chr7 | 6679787 | 6684257 | Peg3 | + |
| chr15 | 72639707 | 72641342 | Peg13 | + |
| chr18 | 13130435 | 13133510 | Impact | + |
| chr7 | 150480736 | 150482810 | KvDMR | + |
| chr7 | 149764673 | 149771930 | Igf2/H19 | + |
| chr8 | 125387861 | 125390344 | Cdh15 | + |
| chr6 | 4696743 | 4699483 | Peg10 | + |
| chr7 | 67147381 | 67151583 | Snrpn | + |
| chr11 | 11925127 | 11927100 | Grb10 | + |
| chr5 | 135825859 | 135825980 | Fkbp6 | + |
| chr7 | 135830870 | 135832249 | Inpp5f_v2 | + |
| chr9 | 89767090 | 89775128 | Rasgrf1 | + |
| chr1 | 63246657 | 63247300 | Gpr1 | - |
| chr2 | 152512421 | 152513169 | Mcts2/H13 | - |
| chr2 | 174150877 | 174154638 | GnasEx1a | - |
| chr1 | 63305566 | 63315360 | Zdbf2 | - |
