# Supplementary material for: Individual retrotransposon integrants are differentially controlled by KZFP/KAP1-dependent histone methylation, DNA methylation and TET-mediated hydroxymethylation in naïve embryonic stem cells
Source: Epigenetics Chromatin. 2018 Feb 26;11:7. doi: 10.1186/s13072-018-0177-1 (PMC6389204; doi:10.1186/s13072-018-0177-1)
Supplement: Supplementary file 11 — Additional file 11. Pattern analysis. [file 13072_2018_177_MOESM11_ESM.zip › Patterns analysis/DataTables/examples/advanced_init/footer_callback.html]

DataTables example - Footer callback


# DataTables example Footer callback

Through the use of the header and footer callback manipulation functions provided by DataTables
(`headerCallbackDT` and `footerCallbackDT`), it is possible to perform
some powerful and useful data manipulation functions, such as summarising data in the table.

The example below shows a footer callback being used to total the data for a column (both the
visible and the hidden data) using the `column().data()DT` API method and `column().footer()DT` for writing the value into the
footer.

| First name | Last name | Position | Office | Salary |
| --- | --- | --- | --- | --- |
| Total: | | | |  |
| --- | --- | --- | --- | --- |
| Tiger | Nixon | System Architect | Edinburgh | $320,800 |
| Garrett | Winters | Accountant | Tokyo | $170,750 |
| Ashton | Cox | Junior Technical Author | San Francisco | $86,000 |
| Cedric | Kelly | Senior Javascript Developer | Edinburgh | $433,060 |
| Airi | Satou | Accountant | Tokyo | $162,700 |
| Brielle | Williamson | Integration Specialist | New York | $372,000 |
| Herrod | Chandler | Sales Assistant | San Francisco | $137,500 |
| Rhona | Davidson | Integration Specialist | Tokyo | $327,900 |
| Colleen | Hurst | Javascript Developer | San Francisco | $205,500 |
| Sonya | Frost | Software Engineer | Edinburgh | $103,600 |
| Jena | Gaines | Office Manager | London | $90,560 |
| Quinn | Flynn | Support Lead | Edinburgh | $342,000 |
| Charde | Marshall | Regional Director | San Francisco | $470,600 |
| Haley | Kennedy | Senior Marketing Designer | London | $313,500 |
| Tatyana | Fitzpatrick | Regional Director | London | $385,750 |
| Michael | Silva | Marketing Designer | London | $198,500 |
| Paul | Byrd | Chief Financial Officer (CFO) | New York | $725,000 |
| Gloria | Little | Systems Administrator | New York | $237,500 |
| Bradley | Greer | Software Engineer | London | $132,000 |
| Dai | Rios | Personnel Lead | Edinburgh | $217,500 |
| Jenette | Caldwell | Development Lead | New York | $345,000 |
| Yuri | Berry | Chief Marketing Officer (CMO) | New York | $675,000 |
| Caesar | Vance | Pre-Sales Support | New York | $106,450 |
| Doris | Wilder | Sales Assistant | Sidney | $85,600 |
| Angelica | Ramos | Chief Executive Officer (CEO) | London | $1,200,000 |
| Gavin | Joyce | Developer | Edinburgh | $92,575 |
| Jennifer | Chang | Regional Director | Singapore | $357,650 |
| Brenden | Wagner | Software Engineer | San Francisco | $206,850 |
| Fiona | Green | Chief Operating Officer (COO) | San Francisco | $850,000 |
| Shou | Itou | Regional Marketing | Tokyo | $163,000 |
| Michelle | House | Integration Specialist | Sidney | $95,400 |
| Suki | Burks | Developer | London | $114,500 |
| Prescott | Bartlett | Technical Author | London | $145,000 |
| Gavin | Cortez | Team Leader | San Francisco | $235,500 |
| Martena | Mccray | Post-Sales support | Edinburgh | $324,050 |
| Unity | Butler | Marketing Designer | San Francisco | $85,675 |
| Howard | Hatfield | Office Manager | San Francisco | $164,500 |
| Hope | Fuentes | Secretary | San Francisco | $109,850 |
| Vivian | Harrell | Financial Controller | San Francisco | $452,500 |
| Timothy | Mooney | Office Manager | London | $136,200 |
| Jackson | Bradshaw | Director | New York | $645,750 |
| Olivia | Liang | Support Engineer | Singapore | $234,500 |
| Bruno | Nash | Software Engineer | London | $163,500 |
| Sakura | Yamamoto | Support Engineer | Tokyo | $139,575 |
| Thor | Walton | Developer | New York | $98,540 |
| Finn | Camacho | Support Engineer | San Francisco | $87,500 |
| Serge | Baldwin | Data Coordinator | Singapore | $138,575 |
| Zenaida | Frank | Software Engineer | New York | $125,250 |
| Zorita | Serrano | Software Engineer | San Francisco | $115,000 |
| Jennifer | Acosta | Junior Javascript Developer | Edinburgh | $75,650 |
| Cara | Stevens | Sales Assistant | New York | $145,600 |
| Hermione | Butler | Regional Director | London | $356,250 |
| Lael | Greer | Systems Administrator | London | $103,500 |
| Jonas | Alexander | Developer | San Francisco | $86,500 |
| Shad | Decker | Regional Director | Edinburgh | $183,000 |
| Michael | Bruce | Javascript Developer | Singapore | $183,000 |
| Donna | Snider | Customer Support | New York | $112,000 |

- Javascript
- HTML
- CSS
- Ajax
- Server-side script

The Javascript shown below is used to initialise the table shown in this
example:

`$(document).ready(function() {
$('#example').dataTable( {
"footerCallback": function ( row, data, start, end, display ) {
var api = this.api(), data;
// Remove the formatting to get integer data for summation
var intVal = function ( i ) {
return typeof i === 'string' ?
i.replace(/[\$,]/g, '')*1 :
typeof i === 'number' ?
i : 0;
};
// Total over all pages
data = api.column( 4 ).data();
total = data.length ?
data.reduce( function (a, b) {
return intVal(a) + intVal(b);
} ) :
0;
// Total over this page
data = api.column( 4, { page: 'current'} ).data();
pageTotal = data.length ?
data.reduce( function (a, b) {
return intVal(a) + intVal(b);
} ) :
0;
// Update footer
$( api.column( 4 ).footer() ).html(
'$'+pageTotal +' ( $'+ total +' total)'
);
}
} );
} );`

In addition to the above code, the following Javascript library files are loaded for use in this
example:

- ../../media/js/jquery.js
- ../../media/js/jquery.dataTables.js

The HTML shown below is the raw HTML table element, before it has been enhanced by
DataTables:

This example uses a little bit of additional CSS beyond what is loaded from the library
files (below), in order to correctly display the table. The additional CSS used is shown
below:

`th { white-space: nowrap; }`

The following CSS library files are loaded for use in this example to provide the styling of the
table:

- ../../media/css/jquery.dataTables.css

This table loads data by Ajax. The latest data that has been loaded is shown below. This data
will update automatically as any additional data is loaded.

The script used to perform the server-side processing for this table is shown below. Please note
that this is just an example script using PHP. Server-side processing scripts can be written in any
language, using the protocol described in the
DataTables documentation.

## Other examples

### Basic initialisation

- Zero configuration
- Feature enable / disable
- Default ordering (sorting)
- Multi-column ordering
- Multiple tables
- Hidden columns
- Complex headers (rowspan and
  colspan)
- DOM positioning
- Flexible table width
- State saving
- Alternative pagination
- Scroll - vertical
- Scroll - horizontal
- Scroll - horizontal and vertical
- Scroll - vertical with jQuery UI
  ThemeRoller
- Language - Comma decimal place
- Language options

### Advanced initialisation

- DOM / jQuery events
- DataTables events
- Column rendering
- Page length options
- Multiple table control elements
- Complex headers (rowspan / colspan)
- Read HTML to data objects
- HTML5 data-\* attributes
- Language file
- Setting defaults
- Row created callback
- Row grouping
- Footer callback
- Custom toolbar elements
- Order direction sequence control

### Styling

- Base style
- Base style - no styling classes
- Base style - cell borders
- Base style - compact
- Base style - hover
- Base style - order-column
- Base style - row borders
- Base style - stripe
- Bootstrap
- Foundation
- jQuery UI ThemeRoller

### Data sources

- HTML (DOM) sourced data
- Ajax sourced data
- Javascript sourced data
- Server-side processing

### API

- Add rows
- Individual column searching (text inputs)
- Individual column searching (select
  inputs)
- Highlighting rows and columns
- Child rows (show extra / detailed
  information)
- Row selection (multiple rows)
- Row selection and deletion (single
  row)
- Form inputs
- Index column
- Show / hide columns dynamically
- Using API in callbacks
- Scrolling and jQuery UI tabs
- Search API (regular expressions)

### Ajax

- Ajax data source (arrays)
- Ajax data source (objects)
- Nested object data (objects)
- Nested object data (arrays)
- Orthogonal data
- Generated content for a column
- Custom data source property
- Flat array data source
- Deferred rendering for speed

### Server-side

- Server-side processing
- Custom HTTP variables
- POST data
- Automatic addition of row ID attributes
- Object data source
- Row details
- Row selection
- JSONP data source for remote domains
- Deferred loading of data
- Pipelining data to reduce Ajax calls for
  paging

### Plug-ins

- API plug-in methods
- Ordering plug-ins (with type
  detection)
- Ordering plug-ins (no type
  detection)
- Custom filtering - range search
- Live DOM ordering

Please refer to the DataTables documentation for full
information about its API properties and methods.  
Additionally, there are a wide range of extras and
plug-ins which extend the capabilities of
DataTables.

DataTables designed and created by SpryMedia Ltd © 2007-2014  
DataTables is licensed under the MIT license.
