# Supplementary material for: Individual retrotransposon integrants are differentially controlled by KZFP/KAP1-dependent histone methylation, DNA methylation and TET-mediated hydroxymethylation in naïve embryonic stem cells
Source: Epigenetics Chromatin. 2018 Feb 26;11:7. doi: 10.1186/s13072-018-0177-1 (PMC6389204; doi:10.1186/s13072-018-0177-1)
Supplement: Supplementary file 11 — Additional file 11. Pattern analysis. [file 13072_2018_177_MOESM11_ESM.zip › Patterns analysis/DataTables/examples/advanced_init/html5-data-attributes.html]

DataTables example - HTML5 data-\* attributes


# DataTables example HTML5 data-\* attributes

DataTables can use different data for different actions (display, ordering and searching) which can
be immensely powerful for transforming data in the display to be intuitive for the end user, while
using different, or more complex data, for other actions. For example, if a table contains a formatted
telephone number in the format `xxx-xxxx`, intuitively a user might search for the number
but without a dash. Using orthogonal data for searching allows both forms of the telephone number to be
used, while only the nicely formatted number is displayed in the table.

One method in which DataTables can obtain this orthogonal data for its different actions is through
custom HTML5 data attributes. DataTables will automatically detect four different attributes on the
HTML elements:

- `data-sort` or `data-order` - for ordering data
- `data-filter` or `data-search` - for search data

This example shows the use of `data-sort` and `data-filter` attributes. In
this case the first column has been formatted so the first name has abbreviated, but the full name is
still searchable (search for "Bruno" for example). Additionally, although the last column contains
non-numeric data in it (`/y`) the column will correctly order numerically as the
`data-sort` / `data-order` attribute is set on the column with plain numeric
data.

| Name | Position | Office | Age | Start date | Salary |
| --- | --- | --- | --- | --- | --- |
| Name | Position | Office | Age | Start date | Salary |
| --- | --- | --- | --- | --- | --- |
| T. Nixon | System Architect | Edinburgh | 61 | Mon 25th Apr 11 | $320,800/y |
| G. Winters | Accountant | Tokyo | 63 | Mon 25th Jul 11 | $170,750/y |
| A. Cox | Junior Technical Author | San Francisco | 66 | Mon 12th Jan 09 | $86,000/y |
| C. Kelly | Senior Javascript Developer | Edinburgh | 22 | Thu 29th Mar 12 | $433,060/y |
| A. Satou | Accountant | Tokyo | 33 | Fri 28th Nov 08 | $162,700/y |
| B. Williamson | Integration Specialist | New York | 61 | Sun 2nd Dec 12 | $372,000/y |
| H. Chandler | Sales Assistant | San Francisco | 59 | Mon 6th Aug 12 | $137,500/y |
| R. Davidson | Integration Specialist | Tokyo | 55 | Thu 14th Oct 10 | $327,900/y |
| C. Hurst | Javascript Developer | San Francisco | 39 | Tue 15th Sep 09 | $205,500/y |
| S. Frost | Software Engineer | Edinburgh | 23 | Sat 13th Dec 08 | $103,600/y |
| J. Gaines | Office Manager | London | 30 | Fri 19th Dec 08 | $90,560/y |
| Q. Flynn | Support Lead | Edinburgh | 22 | Sun 3rd Mar 13 | $342,000/y |
| C. Marshall | Regional Director | San Francisco | 36 | Thu 16th Oct 08 | $470,600/y |
| H. Kennedy | Senior Marketing Designer | London | 43 | Tue 18th Dec 12 | $313,500/y |
| T. Fitzpatrick | Regional Director | London | 19 | Wed 17th Mar 10 | $385,750/y |
| M. Silva | Marketing Designer | London | 66 | Tue 27th Nov 12 | $198,500/y |
| P. Byrd | Chief Financial Officer (CFO) | New York | 64 | Wed 9th Jun 10 | $725,000/y |
| G. Little | Systems Administrator | New York | 59 | Fri 10th Apr 09 | $237,500/y |
| B. Greer | Software Engineer | London | 41 | Sat 13th Oct 12 | $132,000/y |
| D. Rios | Personnel Lead | Edinburgh | 35 | Wed 26th Sep 12 | $217,500/y |
| J. Caldwell | Development Lead | New York | 30 | Sat 3rd Sep 11 | $345,000/y |
| Y. Berry | Chief Marketing Officer (CMO) | New York | 40 | Thu 25th Jun 09 | $675,000/y |
| C. Vance | Pre-Sales Support | New York | 21 | Mon 12th Dec 11 | $106,450/y |
| D. Wilder | Sales Assistant | Sidney | 23 | Mon 20th Sep 10 | $85,600/y |
| A. Ramos | Chief Executive Officer (CEO) | London | 47 | Fri 9th Oct 09 | $1,200,000/y |
| G. Joyce | Developer | Edinburgh | 42 | Wed 22nd Dec 10 | $92,575/y |
| J. Chang | Regional Director | Singapore | 28 | Sun 14th Nov 10 | $357,650/y |
| B. Wagner | Software Engineer | San Francisco | 28 | Tue 7th Jun 11 | $206,850/y |
| F. Green | Chief Operating Officer (COO) | San Francisco | 48 | Thu 11th Mar 10 | $850,000/y |
| S. Itou | Regional Marketing | Tokyo | 20 | Sun 14th Aug 11 | $163,000/y |
| M. House | Integration Specialist | Sidney | 37 | Thu 2nd Jun 11 | $95,400/y |
| S. Burks | Developer | London | 53 | Thu 22nd Oct 09 | $114,500/y |
| P. Bartlett | Technical Author | London | 27 | Sat 7th May 11 | $145,000/y |
| G. Cortez | Team Leader | San Francisco | 22 | Sun 26th Oct 08 | $235,500/y |
| M. Mccray | Post-Sales support | Edinburgh | 46 | Wed 9th Mar 11 | $324,050/y |
| U. Butler | Marketing Designer | San Francisco | 47 | Wed 9th Dec 09 | $85,675/y |
| H. Hatfield | Office Manager | San Francisco | 51 | Tue 16th Dec 08 | $164,500/y |
| H. Fuentes | Secretary | San Francisco | 41 | Fri 12th Feb 10 | $109,850/y |
| V. Harrell | Financial Controller | San Francisco | 62 | Sat 14th Feb 09 | $452,500/y |
| T. Mooney | Office Manager | London | 37 | Thu 11th Dec 08 | $136,200/y |
| J. Bradshaw | Director | New York | 65 | Fri 26th Sep 08 | $645,750/y |
| O. Liang | Support Engineer | Singapore | 64 | Thu 3rd Feb 11 | $234,500/y |
| B. Nash | Software Engineer | London | 38 | Tue 3rd May 11 | $163,500/y |
| S. Yamamoto | Support Engineer | Tokyo | 37 | Wed 19th Aug 09 | $139,575/y |
| T. Walton | Developer | New York | 61 | Sun 11th Aug 13 | $98,540/y |
| F. Camacho | Support Engineer | San Francisco | 47 | Tue 7th Jul 09 | $87,500/y |
| S. Baldwin | Data Coordinator | Singapore | 64 | Mon 9th Apr 12 | $138,575/y |
| Z. Frank | Software Engineer | New York | 63 | Mon 4th Jan 10 | $125,250/y |
| Z. Serrano | Software Engineer | San Francisco | 56 | Fri 1st Jun 12 | $115,000/y |
| J. Acosta | Junior Javascript Developer | Edinburgh | 43 | Fri 1st Feb 13 | $75,650/y |
| C. Stevens | Sales Assistant | New York | 46 | Tue 6th Dec 11 | $145,600/y |
| H. Butler | Regional Director | London | 47 | Mon 21st Mar 11 | $356,250/y |
| L. Greer | Systems Administrator | London | 21 | Fri 27th Feb 09 | $103,500/y |
| J. Alexander | Developer | San Francisco | 30 | Wed 14th Jul 10 | $86,500/y |
| S. Decker | Regional Director | Edinburgh | 51 | Thu 13th Nov 08 | $183,000/y |
| M. Bruce | Javascript Developer | Singapore | 29 | Mon 27th Jun 11 | $183,000/y |
| D. Snider | Customer Support | New York | 27 | Tue 25th Jan 11 | $112,000/y |

- Javascript
- HTML
- CSS
- Ajax
- Server-side script

The Javascript shown below is used to initialise the table shown in this
example:

`$(document).ready(function() {
$('#example').dataTable();
} );`

In addition to the above code, the following Javascript library files are loaded for use in this
example:

- ../../media/js/jquery.js
- ../../media/js/jquery.dataTables.js

The HTML shown below is the raw HTML table element, before it has been enhanced by
DataTables:

This example uses a little bit of additional CSS beyond what is loaded from the library
files (below), in order to correctly display the table. The additional CSS used is shown
below:

The following CSS library files are loaded for use in this example to provide the styling of the
table:

- ../../media/css/jquery.dataTables.css

This table loads data by Ajax. The latest data that has been loaded is shown below. This data
will update automatically as any additional data is loaded.

The script used to perform the server-side processing for this table is shown below. Please note
that this is just an example script using PHP. Server-side processing scripts can be written in any
language, using the protocol described in the
DataTables documentation.

## Other examples

### Basic initialisation

- Zero configuration
- Feature enable / disable
- Default ordering (sorting)
- Multi-column ordering
- Multiple tables
- Hidden columns
- Complex headers (rowspan and
  colspan)
- DOM positioning
- Flexible table width
- State saving
- Alternative pagination
- Scroll - vertical
- Scroll - horizontal
- Scroll - horizontal and vertical
- Scroll - vertical with jQuery UI
  ThemeRoller
- Language - Comma decimal place
- Language options

### Advanced initialisation

- DOM / jQuery events
- DataTables events
- Column rendering
- Page length options
- Multiple table control elements
- Complex headers (rowspan / colspan)
- Read HTML to data objects
- HTML5 data-\* attributes
- Language file
- Setting defaults
- Row created callback
- Row grouping
- Footer callback
- Custom toolbar elements
- Order direction sequence control

### Styling

- Base style
- Base style - no styling classes
- Base style - cell borders
- Base style - compact
- Base style - hover
- Base style - order-column
- Base style - row borders
- Base style - stripe
- Bootstrap
- Foundation
- jQuery UI ThemeRoller

### Data sources

- HTML (DOM) sourced data
- Ajax sourced data
- Javascript sourced data
- Server-side processing

### API

- Add rows
- Individual column searching (text inputs)
- Individual column searching (select
  inputs)
- Highlighting rows and columns
- Child rows (show extra / detailed
  information)
- Row selection (multiple rows)
- Row selection and deletion (single
  row)
- Form inputs
- Index column
- Show / hide columns dynamically
- Using API in callbacks
- Scrolling and jQuery UI tabs
- Search API (regular expressions)

### Ajax

- Ajax data source (arrays)
- Ajax data source (objects)
- Nested object data (objects)
- Nested object data (arrays)
- Orthogonal data
- Generated content for a column
- Custom data source property
- Flat array data source
- Deferred rendering for speed

### Server-side

- Server-side processing
- Custom HTTP variables
- POST data
- Automatic addition of row ID attributes
- Object data source
- Row details
- Row selection
- JSONP data source for remote domains
- Deferred loading of data
- Pipelining data to reduce Ajax calls for
  paging

### Plug-ins

- API plug-in methods
- Ordering plug-ins (with type
  detection)
- Ordering plug-ins (no type
  detection)
- Custom filtering - range search
- Live DOM ordering

Please refer to the DataTables documentation for full
information about its API properties and methods.  
Additionally, there are a wide range of extras and
plug-ins which extend the capabilities of
DataTables.

DataTables designed and created by SpryMedia Ltd © 2007-2014  
DataTables is licensed under the MIT license.
