# Supplementary material for: Individual retrotransposon integrants are differentially controlled by KZFP/KAP1-dependent histone methylation, DNA methylation and TET-mediated hydroxymethylation in naïve embryonic stem cells
Source: Epigenetics Chromatin. 2018 Feb 26;11:7. doi: 10.1186/s13072-018-0177-1 (PMC6389204; doi:10.1186/s13072-018-0177-1)
Supplement: Supplementary file 11 — Additional file 11. Pattern analysis. [file 13072_2018_177_MOESM11_ESM.zip › Patterns analysis/DataTables/examples/ajax/simple.html]

DataTables example - Ajax data source (arrays)


# DataTables example Ajax data source (arrays)

DataTables has the ability to read data from virtually any JSON data source that can be obtained by
Ajax. This can be done, in its most simple form, by setting the `ajaxDT` option to the address of the JSON
data source.

The `ajaxDT` option also allows for more advanced
configuration such as altering how the Ajax request is made. See the `ajaxDT` documentation or the other Ajax
examples for DataTables for further information.

By default DataTables will assume that an array data source is to be used and will read the
information to be displayed in each column from the row's array using the column index, making working
with arrays very simple (note that this can be changed, or objects used may using the `columns.dataDT` option, shown in other
examples).

The example below shows DataTables loading data for a table from arrays as the data source, where
the structure of the row's data source in this example is:

```
[
	"Tiger Nixon",
	"System Architect",
	"Edinburgh",
	"5421",
	"2011/04/25",
	"$3,120"
]
```

| Name | Position | Office | Extn. | Start date | Salary |
| --- | --- | --- | --- | --- | --- |
| Name | Position | Office | Extn. | Start date | Salary |
| --- | --- | --- | --- | --- | --- |

- Javascript
- HTML
- CSS
- Ajax
- Server-side script

The Javascript shown below is used to initialise the table shown in this
example:

`$(document).ready(function() {
$('#example').dataTable( {
"ajax": "data/arrays.txt"
} );
} );`

In addition to the above code, the following Javascript library files are loaded for use in this
example:

- ../../media/js/jquery.js
- ../../media/js/jquery.dataTables.js

The HTML shown below is the raw HTML table element, before it has been enhanced by
DataTables:

This example uses a little bit of additional CSS beyond what is loaded from the library
files (below), in order to correctly display the table. The additional CSS used is shown
below:

The following CSS library files are loaded for use in this example to provide the styling of the
table:

- ../../media/css/jquery.dataTables.css

This table loads data by Ajax. The latest data that has been loaded is shown below. This data
will update automatically as any additional data is loaded.

The script used to perform the server-side processing for this table is shown below. Please note
that this is just an example script using PHP. Server-side processing scripts can be written in any
language, using the protocol described in the
DataTables documentation.

## Other examples

### Basic initialisation

- Zero configuration
- Feature enable / disable
- Default ordering (sorting)
- Multi-column ordering
- Multiple tables
- Hidden columns
- Complex headers (rowspan and
  colspan)
- DOM positioning
- Flexible table width
- State saving
- Alternative pagination
- Scroll - vertical
- Scroll - horizontal
- Scroll - horizontal and vertical
- Scroll - vertical with jQuery UI
  ThemeRoller
- Language - Comma decimal place
- Language options

### Advanced initialisation

- DOM / jQuery events
- DataTables events
- Column rendering
- Page length options
- Multiple table control
  elements
- Complex headers (rowspan /
  colspan)
- Read HTML to data objects
- HTML5 data-\* attributes
- Language file
- Setting defaults
- Row created callback
- Row grouping
- Footer callback
- Custom toolbar elements
- Order direction sequence
  control

### Styling

- Base style
- Base style - no styling classes
- Base style - cell borders
- Base style - compact
- Base style - hover
- Base style - order-column
- Base style - row borders
- Base style - stripe
- Bootstrap
- Foundation
- jQuery UI ThemeRoller

### Data sources

- HTML (DOM) sourced data
- Ajax sourced data
- Javascript sourced data
- Server-side processing

### API

- Add rows
- Individual column searching (text inputs)
- Individual column searching (select
  inputs)
- Highlighting rows and columns
- Child rows (show extra / detailed
  information)
- Row selection (multiple rows)
- Row selection and deletion (single
  row)
- Form inputs
- Index column
- Show / hide columns dynamically
- Using API in callbacks
- Scrolling and jQuery UI tabs
- Search API (regular expressions)

### Ajax

- Ajax data source (arrays)
- Ajax data source (objects)
- Nested object data (objects)
- Nested object data (arrays)
- Orthogonal data
- Generated content for a column
- Custom data source property
- Flat array data source
- Deferred rendering for speed

### Server-side

- Server-side processing
- Custom HTTP variables
- POST data
- Automatic addition of row ID attributes
- Object data source
- Row details
- Row selection
- JSONP data source for remote domains
- Deferred loading of data
- Pipelining data to reduce Ajax calls for
  paging

### Plug-ins

- API plug-in methods
- Ordering plug-ins (with type
  detection)
- Ordering plug-ins (no type
  detection)
- Custom filtering - range search
- Live DOM ordering

Please refer to the DataTables documentation for full
information about its API properties and methods.  
Additionally, there are a wide range of extras and
plug-ins which extend the capabilities of
DataTables.

DataTables designed and created by SpryMedia Ltd © 2007-2014  
DataTables is licensed under the MIT license.
