# Supplementary material for: Individual retrotransposon integrants are differentially controlled by KZFP/KAP1-dependent histone methylation, DNA methylation and TET-mediated hydroxymethylation in naïve embryonic stem cells
Source: Epigenetics Chromatin. 2018 Feb 26;11:7. doi: 10.1186/s13072-018-0177-1 (PMC6389204; doi:10.1186/s13072-018-0177-1)
Supplement: Supplementary file 11 — Additional file 11. Pattern analysis. [file 13072_2018_177_MOESM11_ESM.zip › Patterns analysis/DataTables/examples/api/form.html]

DataTables example - Form inputs


# DataTables example Form inputs

In order to perform paging, ordering, searching etc, DataTables can remove rows and cells from the
document (i.e. those rows / cells which are not needed are not inserted into the document). This
increases performance and compatibility, however, it means that submitting forms which span multiple
pages requires a little bit of additional work to get the information that is not in the document any
longer.

The `$()DT` method can be used to get nodes from the document
regardless of paging, ordering etc. This example shows `$()DT` being used to get all `input` elements from the table.

In the example a simple `alert()` is used to show the information from the form, but an
Ajax call to the server with the form data could easily be performed.

Submit form

| Name | Age | Position | Office |
| --- | --- | --- | --- |
| Name | Age | Position | Office |
| --- | --- | --- | --- |
| Tiger Nixon |  |  | Edinburgh  London  New York  San Francisco  Tokyo |
| Garrett Winters |  |  | Edinburgh  London  New York  San Francisco  Tokyo |
| Ashton Cox |  |  | Edinburgh  London  New York  San Francisco  Tokyo |
| Cedric Kelly |  |  | Edinburgh  London  New York  San Francisco  Tokyo |
| Airi Satou |  |  | Edinburgh  London  New York  San Francisco  Tokyo |
| Brielle Williamson |  |  | Edinburgh  London  New York  San Francisco  Tokyo |
| Herrod Chandler |  |  | Edinburgh  London  New York  San Francisco  Tokyo |
| Rhona Davidson |  |  | Edinburgh  London  New York  San Francisco  Tokyo |
| Colleen Hurst |  |  | Edinburgh  London  New York  San Francisco  Tokyo |
| Sonya Frost |  |  | Edinburgh  London  New York  San Francisco  Tokyo |
| Jena Gaines |  |  | Edinburgh  London  New York  San Francisco  Tokyo |
| Quinn Flynn |  |  | Edinburgh  London  New York  San Francisco  Tokyo |
| Charde Marshall |  |  | Edinburgh  London  New York  San Francisco  Tokyo |
| Haley Kennedy |  |  | Edinburgh  London  New York  San Francisco  Tokyo |
| Tatyana Fitzpatrick |  |  | Edinburgh  London  New York  San Francisco  Tokyo |
| Michael Silva |  |  | Edinburgh  London  New York  San Francisco  Tokyo |
| Paul Byrd |  |  | Edinburgh  London  New York  San Francisco  Tokyo |
| Gloria Little |  |  | Edinburgh  London  New York  San Francisco  Tokyo |
| Bradley Greer |  |  | Edinburgh  London  New York  San Francisco  Tokyo |
| Dai Rios |  |  | Edinburgh  London  New York  San Francisco  Tokyo |
| Jenette Caldwell |  |  | Edinburgh  London  New York  San Francisco  Tokyo |
| Yuri Berry |  |  | Edinburgh  London  New York  San Francisco  Tokyo |
| Caesar Vance |  |  | Edinburgh  London  New York  San Francisco  Tokyo |
| Doris Wilder |  |  | Edinburgh  London  New York  San Francisco  Tokyo |
| Angelica Ramos |  |  | Edinburgh  London  New York  San Francisco  Tokyo |
| Gavin Joyce |  |  | Edinburgh  London  New York  San Francisco  Tokyo |
| Jennifer Chang |  |  | Edinburgh  London  New York  San Francisco  Tokyo |
| Brenden Wagner |  |  | Edinburgh  London  New York  San Francisco  Tokyo |
| Fiona Green |  |  | Edinburgh  London  New York  San Francisco  Tokyo |
| Shou Itou |  |  | Edinburgh  London  New York  San Francisco  Tokyo |
| Michelle House |  |  | Edinburgh  London  New York  San Francisco  Tokyo |
| Suki Burks |  |  | Edinburgh  London  New York  San Francisco  Tokyo |
| Prescott Bartlett |  |  | Edinburgh  London  New York  San Francisco  Tokyo |
| Gavin Cortez |  |  | Edinburgh  London  New York  San Francisco  Tokyo |
| Martena Mccray |  |  | Edinburgh  London  New York  San Francisco  Tokyo |
| Unity Butler |  |  | Edinburgh  London  New York  San Francisco  Tokyo |
| Howard Hatfield |  |  | Edinburgh  London  New York  San Francisco  Tokyo |
| Hope Fuentes |  |  | Edinburgh  London  New York  San Francisco  Tokyo |
| Vivian Harrell |  |  | Edinburgh  London  New York  San Francisco  Tokyo |
| Timothy Mooney |  |  | Edinburgh  London  New York  San Francisco  Tokyo |
| Jackson Bradshaw |  |  | Edinburgh  London  New York  San Francisco  Tokyo |
| Olivia Liang |  |  | Edinburgh  London  New York  San Francisco  Tokyo |
| Bruno Nash |  |  | Edinburgh  London  New York  San Francisco  Tokyo |
| Sakura Yamamoto |  |  | Edinburgh  London  New York  San Francisco  Tokyo |
| Thor Walton |  |  | Edinburgh  London  New York  San Francisco  Tokyo |
| Finn Camacho |  |  | Edinburgh  London  New York  San Francisco  Tokyo |
| Serge Baldwin |  |  | Edinburgh  London  New York  San Francisco  Tokyo |
| Zenaida Frank |  |  | Edinburgh  London  New York  San Francisco  Tokyo |
| Zorita Serrano |  |  | Edinburgh  London  New York  San Francisco  Tokyo |
| Jennifer Acosta |  |  | Edinburgh  London  New York  San Francisco  Tokyo |
| Cara Stevens |  |  | Edinburgh  London  New York  San Francisco  Tokyo |
| Hermione Butler |  |  | Edinburgh  London  New York  San Francisco  Tokyo |
| Lael Greer |  |  | Edinburgh  London  New York  San Francisco  Tokyo |
| Jonas Alexander |  |  | Edinburgh  London  New York  San Francisco  Tokyo |
| Shad Decker |  |  | Edinburgh  London  New York  San Francisco  Tokyo |
| Michael Bruce |  |  | Edinburgh  London  New York  San Francisco  Tokyo |
| Donna Snider |  |  | Edinburgh  London  New York  San Francisco  Tokyo |

- Javascript
- HTML
- CSS
- Ajax
- Server-side script

The Javascript shown below is used to initialise the table shown in this
example:

`$(document).ready(function() {
var table = $('#example').DataTable();
$('button').click( function() {
var data = table.$('input, select').serialize();
alert(
"The following data would have been submitted to the server: \n\n"+
data.substr( 0, 120 )+'...'
);
return false;
} );
} );`

In addition to the above code, the following Javascript library files are loaded for use in this
example:

- ../../media/js/jquery.js
- ../../media/js/jquery.dataTables.js

The HTML shown below is the raw HTML table element, before it has been enhanced by
DataTables:

This example uses a little bit of additional CSS beyond what is loaded from the library
files (below), in order to correctly display the table. The additional CSS used is shown
below:

The following CSS library files are loaded for use in this example to provide the styling of the
table:

- ../../media/css/jquery.dataTables.css

This table loads data by Ajax. The latest data that has been loaded is shown below. This data
will update automatically as any additional data is loaded.

The script used to perform the server-side processing for this table is shown below. Please note
that this is just an example script using PHP. Server-side processing scripts can be written in any
language, using the protocol described in the
DataTables documentation.

## Other examples

### Basic initialisation

- Zero configuration
- Feature enable / disable
- Default ordering (sorting)
- Multi-column ordering
- Multiple tables
- Hidden columns
- Complex headers (rowspan and
  colspan)
- DOM positioning
- Flexible table width
- State saving
- Alternative pagination
- Scroll - vertical
- Scroll - horizontal
- Scroll - horizontal and vertical
- Scroll - vertical with jQuery UI
  ThemeRoller
- Language - Comma decimal place
- Language options

### Advanced initialisation

- DOM / jQuery events
- DataTables events
- Column rendering
- Page length options
- Multiple table control
  elements
- Complex headers (rowspan /
  colspan)
- Read HTML to data objects
- HTML5 data-\* attributes
- Language file
- Setting defaults
- Row created callback
- Row grouping
- Footer callback
- Custom toolbar elements
- Order direction sequence
  control

### Styling

- Base style
- Base style - no styling classes
- Base style - cell borders
- Base style - compact
- Base style - hover
- Base style - order-column
- Base style - row borders
- Base style - stripe
- Bootstrap
- Foundation
- jQuery UI ThemeRoller

### Data sources

- HTML (DOM) sourced data
- Ajax sourced data
- Javascript sourced data
- Server-side processing

### API

- Add rows
- Individual column searching (text inputs)
- Individual column searching (select
  inputs)
- Highlighting rows and columns
- Child rows (show extra / detailed information)
- Row selection (multiple rows)
- Row selection and deletion (single row)
- Form inputs
- Index column
- Show / hide columns dynamically
- Using API in callbacks
- Scrolling and jQuery UI tabs
- Search API (regular expressions)

### Ajax

- Ajax data source (arrays)
- Ajax data source (objects)
- Nested object data (objects)
- Nested object data (arrays)
- Orthogonal data
- Generated content for a column
- Custom data source property
- Flat array data source
- Deferred rendering for speed

### Server-side

- Server-side processing
- Custom HTTP variables
- POST data
- Automatic addition of row ID attributes
- Object data source
- Row details
- Row selection
- JSONP data source for remote domains
- Deferred loading of data
- Pipelining data to reduce Ajax calls for
  paging

### Plug-ins

- API plug-in methods
- Ordering plug-ins (with type
  detection)
- Ordering plug-ins (no type
  detection)
- Custom filtering - range search
- Live DOM ordering

Please refer to the DataTables documentation for full
information about its API properties and methods.  
Additionally, there are a wide range of extras and
plug-ins which extend the capabilities of
DataTables.

DataTables designed and created by SpryMedia Ltd © 2007-2014  
DataTables is licensed under the MIT license.
