# Supplementary material for: Individual retrotransposon integrants are differentially controlled by KZFP/KAP1-dependent histone methylation, DNA methylation and TET-mediated hydroxymethylation in naïve embryonic stem cells
Source: Epigenetics Chromatin. 2018 Feb 26;11:7. doi: 10.1186/s13072-018-0177-1 (PMC6389204; doi:10.1186/s13072-018-0177-1)
Supplement: Supplementary file 11 — Additional file 11. Pattern analysis. [file 13072_2018_177_MOESM11_ESM.zip › Patterns analysis/DataTables/examples/data_sources/index.html]

DataTables examples - Data sources


# DataTables example Data sources

DataTables can obtain data from four different fundamental sources:

- HTML document (DOM)
- Javascript (array / objects)
- Ajax sourced data with client-side processing
- Ajax sourced data with server-side processing

Which of these options is used to populate the table with data depends upon how the table is
initialised. The examples in this section show these four different data source types.

### Data sources

- HTML (DOM) sourced data
- Ajax sourced data
- Javascript sourced data
- Server-side processing

Please refer to the DataTables documentation for full
information about its API properties and methods.  
Additionally, there are a wide range of extras and
plug-ins which extend the capabilities of
DataTables.

DataTables designed and created by SpryMedia Ltd © 2007-2014  
DataTables is licensed under the MIT license.
