# Supplementary material for: Individual retrotransposon integrants are differentially controlled by KZFP/KAP1-dependent histone methylation, DNA methylation and TET-mediated hydroxymethylation in naïve embryonic stem cells
Source: Epigenetics Chromatin. 2018 Feb 26;11:7. doi: 10.1186/s13072-018-0177-1 (PMC6389204; doi:10.1186/s13072-018-0177-1)
Supplement: Supplementary file 11 — Additional file 11. Pattern analysis. [file 13072_2018_177_MOESM11_ESM.zip › Patterns analysis/DataTables/examples/data_sources/js_array.html]

DataTables example - Javascript sourced data


# DataTables example Javascript sourced data

At times you will wish to be able to create a table from dynamic information passed directly to
DataTables, rather than having it read from the document. This is achieved using the `dataDT` option in the initialisation object,
passing in an array of data to be used (like all other DataTables handled data, this can be arrays or
objects using the `columns.dataDT` option).

A `<table>` must be available on the page for DataTables to use. This examples
shows the element being added by Javascript and then initialising the DataTable with a set of data from
a Javascript array.

- Javascript
- HTML
- CSS
- Ajax
- Server-side script

The Javascript shown below is used to initialise the table shown in this
example:

`var dataSet = [
['Trident','Internet Explorer 4.0','Win 95+','4','X'],
['Trident','Internet Explorer 5.0','Win 95+','5','C'],
['Trident','Internet Explorer 5.5','Win 95+','5.5','A'],
['Trident','Internet Explorer 6','Win 98+','6','A'],
['Trident','Internet Explorer 7','Win XP SP2+','7','A'],
['Trident','AOL browser (AOL desktop)','Win XP','6','A'],
['Gecko','Firefox 1.0','Win 98+ / OSX.2+','1.7','A'],
['Gecko','Firefox 1.5','Win 98+ / OSX.2+','1.8','A'],
['Gecko','Firefox 2.0','Win 98+ / OSX.2+','1.8','A'],
['Gecko','Firefox 3.0','Win 2k+ / OSX.3+','1.9','A'],
['Gecko','Camino 1.0','OSX.2+','1.8','A'],
['Gecko','Camino 1.5','OSX.3+','1.8','A'],
['Gecko','Netscape 7.2','Win 95+ / Mac OS 8.6-9.2','1.7','A'],
['Gecko','Netscape Browser 8','Win 98SE+','1.7','A'],
['Gecko','Netscape Navigator 9','Win 98+ / OSX.2+','1.8','A'],
['Gecko','Mozilla 1.0','Win 95+ / OSX.1+',1,'A'],
['Gecko','Mozilla 1.1','Win 95+ / OSX.1+',1.1,'A'],
['Gecko','Mozilla 1.2','Win 95+ / OSX.1+',1.2,'A'],
['Gecko','Mozilla 1.3','Win 95+ / OSX.1+',1.3,'A'],
['Gecko','Mozilla 1.4','Win 95+ / OSX.1+',1.4,'A'],
['Gecko','Mozilla 1.5','Win 95+ / OSX.1+',1.5,'A'],
['Gecko','Mozilla 1.6','Win 95+ / OSX.1+',1.6,'A'],
['Gecko','Mozilla 1.7','Win 98+ / OSX.1+',1.7,'A'],
['Gecko','Mozilla 1.8','Win 98+ / OSX.1+',1.8,'A'],
['Gecko','Seamonkey 1.1','Win 98+ / OSX.2+','1.8','A'],
['Gecko','Epiphany 2.20','Gnome','1.8','A'],
['Webkit','Safari 1.2','OSX.3','125.5','A'],
['Webkit','Safari 1.3','OSX.3','312.8','A'],
['Webkit','Safari 2.0','OSX.4+','419.3','A'],
['Webkit','Safari 3.0','OSX.4+','522.1','A'],
['Webkit','OmniWeb 5.5','OSX.4+','420','A'],
['Webkit','iPod Touch / iPhone','iPod','420.1','A'],
['Webkit','S60','S60','413','A'],
['Presto','Opera 7.0','Win 95+ / OSX.1+','-','A'],
['Presto','Opera 7.5','Win 95+ / OSX.2+','-','A'],
['Presto','Opera 8.0','Win 95+ / OSX.2+','-','A'],
['Presto','Opera 8.5','Win 95+ / OSX.2+','-','A'],
['Presto','Opera 9.0','Win 95+ / OSX.3+','-','A'],
['Presto','Opera 9.2','Win 88+ / OSX.3+','-','A'],
['Presto','Opera 9.5','Win 88+ / OSX.3+','-','A'],
['Presto','Opera for Wii','Wii','-','A'],
['Presto','Nokia N800','N800','-','A'],
['Presto','Nintendo DS browser','Nintendo DS','8.5','C/A<sup>1</sup>'],
['KHTML','Konqureror 3.1','KDE 3.1','3.1','C'],
['KHTML','Konqureror 3.3','KDE 3.3','3.3','A'],
['KHTML','Konqureror 3.5','KDE 3.5','3.5','A'],
['Tasman','Internet Explorer 4.5','Mac OS 8-9','-','X'],
['Tasman','Internet Explorer 5.1','Mac OS 7.6-9','1','C'],
['Tasman','Internet Explorer 5.2','Mac OS 8-X','1','C'],
['Misc','NetFront 3.1','Embedded devices','-','C'],
['Misc','NetFront 3.4','Embedded devices','-','A'],
['Misc','Dillo 0.8','Embedded devices','-','X'],
['Misc','Links','Text only','-','X'],
['Misc','Lynx','Text only','-','X'],
['Misc','IE Mobile','Windows Mobile 6','-','C'],
['Misc','PSP browser','PSP','-','C'],
['Other browsers','All others','-','-','U']
];
$(document).ready(function() {
$('#demo').html( '<table cellpadding="0" cellspacing="0" border="0" class="display" id="example"></table>' );
$('#example').dataTable( {
"data": dataSet,
"columns": [
{ "title": "Engine" },
{ "title": "Browser" },
{ "title": "Platform" },
{ "title": "Version", "class": "center" },
{ "title": "Grade", "class": "center" }
]
} );
} );`

In addition to the above code, the following Javascript library files are loaded for use in this
example:

- ../../media/js/jquery.js
- ../../media/js/jquery.dataTables.js

The HTML shown below is the raw HTML table element, before it has been enhanced by
DataTables:

This example uses a little bit of additional CSS beyond what is loaded from the library
files (below), in order to correctly display the table. The additional CSS used is shown
below:

The following CSS library files are loaded for use in this example to provide the styling of the
table:

- ../../media/css/jquery.dataTables.css

This table loads data by Ajax. The latest data that has been loaded is shown below. This data
will update automatically as any additional data is loaded.

The script used to perform the server-side processing for this table is shown below. Please note
that this is just an example script using PHP. Server-side processing scripts can be written in any
language, using the protocol described in the
DataTables documentation.

## Other examples

### Basic initialisation

- Zero configuration
- Feature enable / disable
- Default ordering (sorting)
- Multi-column ordering
- Multiple tables
- Hidden columns
- Complex headers (rowspan and
  colspan)
- DOM positioning
- Flexible table width
- State saving
- Alternative pagination
- Scroll - vertical
- Scroll - horizontal
- Scroll - horizontal and vertical
- Scroll - vertical with jQuery UI
  ThemeRoller
- Language - Comma decimal place
- Language options

### Advanced initialisation

- DOM / jQuery events
- DataTables events
- Column rendering
- Page length options
- Multiple table control
  elements
- Complex headers (rowspan /
  colspan)
- Read HTML to data objects
- HTML5 data-\* attributes
- Language file
- Setting defaults
- Row created callback
- Row grouping
- Footer callback
- Custom toolbar elements
- Order direction sequence
  control

### Styling

- Base style
- Base style - no styling classes
- Base style - cell borders
- Base style - compact
- Base style - hover
- Base style - order-column
- Base style - row borders
- Base style - stripe
- Bootstrap
- Foundation
- jQuery UI ThemeRoller

### Data sources

- HTML (DOM) sourced data
- Ajax sourced data
- Javascript sourced data
- Server-side processing

### API

- Add rows
- Individual column searching (text inputs)
- Individual column searching (select
  inputs)
- Highlighting rows and columns
- Child rows (show extra / detailed
  information)
- Row selection (multiple rows)
- Row selection and deletion (single
  row)
- Form inputs
- Index column
- Show / hide columns dynamically
- Using API in callbacks
- Scrolling and jQuery UI tabs
- Search API (regular expressions)

### Ajax

- Ajax data source (arrays)
- Ajax data source (objects)
- Nested object data (objects)
- Nested object data (arrays)
- Orthogonal data
- Generated content for a column
- Custom data source property
- Flat array data source
- Deferred rendering for speed

### Server-side

- Server-side processing
- Custom HTTP variables
- POST data
- Automatic addition of row ID attributes
- Object data source
- Row details
- Row selection
- JSONP data source for remote domains
- Deferred loading of data
- Pipelining data to reduce Ajax calls for
  paging

### Plug-ins

- API plug-in methods
- Ordering plug-ins (with type
  detection)
- Ordering plug-ins (no type
  detection)
- Custom filtering - range search
- Live DOM ordering

Please refer to the DataTables documentation for full
information about its API properties and methods.  
Additionally, there are a wide range of extras and
plug-ins which extend the capabilities of
DataTables.

DataTables designed and created by SpryMedia Ltd © 2007-2014  
DataTables is licensed under the MIT license.
