# Supplementary material for: Individual retrotransposon integrants are differentially controlled by KZFP/KAP1-dependent histone methylation, DNA methylation and TET-mediated hydroxymethylation in naïve embryonic stem cells
Source: Epigenetics Chromatin. 2018 Feb 26;11:7. doi: 10.1186/s13072-018-0177-1 (PMC6389204; doi:10.1186/s13072-018-0177-1)
Supplement: Supplementary file 11 — Additional file 11. Pattern analysis. [file 13072_2018_177_MOESM11_ESM.zip › Patterns analysis/DataTables/examples/plug-ins/index.html]

DataTables examples - Plug-ins


# DataTables example Plug-ins

While DataTables has a wide range of options and data type support built in, it can never cater for
every type of data out of the box. For this reason, DataTables exposes an extension API which allows
you, the developers using DataTables, to add support for your own data types, searching, ordering and
feature plug-ins.

The examples in this section show how plug-ins can be used and developed for DataTables.

### Plug-ins

- API plug-in methods
- Ordering plug-ins (with type detection)
- Ordering plug-ins (no type detection)
- Custom filtering - range search
- Live DOM ordering

Please refer to the DataTables documentation for full
information about its API properties and methods.  
Additionally, there are a wide range of extras and
plug-ins which extend the capabilities of
DataTables.

DataTables designed and created by SpryMedia Ltd © 2007-2014  
DataTables is licensed under the MIT license.
