# Supplementary material for: Individual retrotransposon integrants are differentially controlled by KZFP/KAP1-dependent histone methylation, DNA methylation and TET-mediated hydroxymethylation in naïve embryonic stem cells
Source: Epigenetics Chromatin. 2018 Feb 26;11:7. doi: 10.1186/s13072-018-0177-1 (PMC6389204; doi:10.1186/s13072-018-0177-1)
Supplement: Supplementary file 11 — Additional file 11. Pattern analysis. [file 13072_2018_177_MOESM11_ESM.zip › Patterns analysis/DataTables/examples/plug-ins/sorting_auto.html]

DataTables example - Ordering plug-ins (with type detection)


# DataTables example Ordering plug-ins (with type detection)

Although DataTables will automatically order data from a number of different data types using the
built in methods, When dealing with more complex formatted data, it can be desirable to define the
ordering order yourself. Using plug-in ordering functions, you have have DataTables sort data in any
manner you wish.

Formatted data of a particular kind can be automatically detected and a suitable ordering plug-in
assigned to it by making use of DataTables' plug-in type detection abilities. For complete information
about type detection and ordering plug-ins; creating them and their requirements, please refer to the
plug-in development documentation.

This example shows ordering with using an enumerated type.

A wide variety of ready made ordering plug-ins can be found on the DataTables plug-ins page.

| Name | Position | Office | Age | Start date | Salary |
| --- | --- | --- | --- | --- | --- |
| Name | Position | Office | Age | Start date | Salary |
| --- | --- | --- | --- | --- | --- |
| Tiger Nixon | System Architect | Edinburgh | 61 | 2011/04/25 | Low |
| Garrett Winters | Accountant | Tokyo | 63 | 2011/07/25 | Low |
| Ashton Cox | Junior Technical Author | San Francisco | 66 | 2009/01/12 | Low |
| Cedric Kelly | Senior Javascript Developer | Edinburgh | 22 | 2012/03/29 | Medium |
| Airi Satou | Accountant | Tokyo | 33 | 2008/11/28 | Low |
| Brielle Williamson | Integration Specialist | New York | 61 | 2012/12/02 | Medium |
| Herrod Chandler | Sales Assistant | San Francisco | 59 | 2012/08/06 | Low |
| Rhona Davidson | Integration Specialist | Tokyo | 55 | 2010/10/14 | Low |
| Colleen Hurst | Javascript Developer | San Francisco | 39 | 2009/09/15 | Low |
| Sonya Frost | Software Engineer | Edinburgh | 23 | 2008/12/13 | Low |
| Jena Gaines | Office Manager | London | 30 | 2008/12/19 | Low |
| Quinn Flynn | Support Lead | Edinburgh | 22 | 2013/03/03 | Low |
| Charde Marshall | Regional Director | San Francisco | 36 | 2008/10/16 | Medium |
| Haley Kennedy | Senior Marketing Designer | London | 43 | 2012/12/18 | Low |
| Tatyana Fitzpatrick | Regional Director | London | 19 | 2010/03/17 | Medium |
| Michael Silva | Marketing Designer | London | 66 | 2012/11/27 | Low |
| Paul Byrd | Chief Financial Officer (CFO) | New York | 64 | 2010/06/09 | High |
| Gloria Little | Systems Administrator | New York | 59 | 2009/04/10 | Low |
| Bradley Greer | Software Engineer | London | 41 | 2012/10/13 | Low |
| Dai Rios | Personnel Lead | Edinburgh | 35 | 2012/09/26 | Low |
| Jenette Caldwell | Development Lead | New York | 30 | 2011/09/03 | Low |
| Yuri Berry | Chief Marketing Officer (CMO) | New York | 40 | 2009/06/25 | High |
| Caesar Vance | Pre-Sales Support | New York | 21 | 2011/12/12 | Low |
| Doris Wilder | Sales Assistant | Sidney | 23 | 2010/09/20 | Low |
| Angelica Ramos | Chief Executive Officer (CEO) | London | 47 | 2009/10/09 | High |
| Gavin Joyce | Developer | Edinburgh | 42 | 2010/12/22 | Low |
| Jennifer Chang | Regional Director | Singapore | 28 | 2010/11/14 | Medium |
| Brenden Wagner | Software Engineer | San Francisco | 28 | 2011/06/07 | Low |
| Fiona Green | Chief Operating Officer (COO) | San Francisco | 48 | 2010/03/11 | High |
| Shou Itou | Regional Marketing | Tokyo | 20 | 2011/08/14 | Low |
| Michelle House | Integration Specialist | Sidney | 37 | 2011/06/02 | Low |
| Suki Burks | Developer | London | 53 | 2009/10/22 | Low |
| Prescott Bartlett | Technical Author | London | 27 | 2011/05/07 | Low |
| Gavin Cortez | Team Leader | San Francisco | 22 | 2008/10/26 | Low |
| Martena Mccray | Post-Sales support | Edinburgh | 46 | 2011/03/09 | Low |
| Unity Butler | Marketing Designer | San Francisco | 47 | 2009/12/09 | Low |
| Howard Hatfield | Office Manager | San Francisco | 51 | 2008/12/16 | Low |
| Hope Fuentes | Secretary | San Francisco | 41 | 2010/02/12 | Low |
| Vivian Harrell | Financial Controller | San Francisco | 62 | 2009/02/14 | Medium |
| Timothy Mooney | Office Manager | London | 37 | 2008/12/11 | Low |
| Jackson Bradshaw | Director | New York | 65 | 2008/09/26 | Medium |
| Olivia Liang | Support Engineer | Singapore | 64 | 2011/02/03 | Low |
| Bruno Nash | Software Engineer | London | 38 | 2011/05/03 | Low |
| Sakura Yamamoto | Support Engineer | Tokyo | 37 | 2009/08/19 | Low |
| Thor Walton | Developer | New York | 61 | 2013/08/11 | Low |
| Finn Camacho | Support Engineer | San Francisco | 47 | 2009/07/07 | Low |
| Serge Baldwin | Data Coordinator | Singapore | 64 | 2012/04/09 | Low |
| Zenaida Frank | Software Engineer | New York | 63 | 2010/01/04 | Low |
| Zorita Serrano | Software Engineer | San Francisco | 56 | 2012/06/01 | Low |
| Jennifer Acosta | Junior Javascript Developer | Edinburgh | 43 | 2013/02/01 | Low |
| Cara Stevens | Sales Assistant | New York | 46 | 2011/12/06 | Low |
| Hermione Butler | Regional Director | London | 47 | 2011/03/21 | Medium |
| Lael Greer | Systems Administrator | London | 21 | 2009/02/27 | Low |
| Jonas Alexander | Developer | San Francisco | 30 | 2010/07/14 | Low |
| Shad Decker | Regional Director | Edinburgh | 51 | 2008/11/13 | Low |
| Michael Bruce | Javascript Developer | Singapore | 29 | 2011/06/27 | Low |
| Donna Snider | Customer Support | New York | 27 | 2011/01/25 | Low |

- Javascript
- HTML
- CSS
- Ajax
- Server-side script

The Javascript shown below is used to initialise the table shown in this
example:

`$.fn.dataTable.ext.type.detect.unshift(
function ( d ) {
return d === 'Low' || d === 'Medium' || d === 'High' ?
'salary-grade' :
null;
}
);
$.fn.dataTable.ext.type.order['salary-grade-pre'] = function ( d ) {
switch ( d ) {
case 'Low': return 1;
case 'Medium': return 2;
case 'High': return 3;
}
return 0;
};
$(document).ready(function() {
$('#example').dataTable();
} );`

In addition to the above code, the following Javascript library files are loaded for use in this
example:

- ../../media/js/jquery.js
- ../../media/js/jquery.dataTables.js

The HTML shown below is the raw HTML table element, before it has been enhanced by
DataTables:

This example uses a little bit of additional CSS beyond what is loaded from the library
files (below), in order to correctly display the table. The additional CSS used is shown
below:

The following CSS library files are loaded for use in this example to provide the styling of the
table:

- ../../media/css/jquery.dataTables.css

This table loads data by Ajax. The latest data that has been loaded is shown below. This data
will update automatically as any additional data is loaded.

The script used to perform the server-side processing for this table is shown below. Please note
that this is just an example script using PHP. Server-side processing scripts can be written in any
language, using the protocol described in the
DataTables documentation.

## Other examples

### Basic initialisation

- Zero configuration
- Feature enable / disable
- Default ordering (sorting)
- Multi-column ordering
- Multiple tables
- Hidden columns
- Complex headers (rowspan and
  colspan)
- DOM positioning
- Flexible table width
- State saving
- Alternative pagination
- Scroll - vertical
- Scroll - horizontal
- Scroll - horizontal and vertical
- Scroll - vertical with jQuery UI
  ThemeRoller
- Language - Comma decimal place
- Language options

### Advanced initialisation

- DOM / jQuery events
- DataTables events
- Column rendering
- Page length options
- Multiple table control
  elements
- Complex headers (rowspan /
  colspan)
- Read HTML to data objects
- HTML5 data-\* attributes
- Language file
- Setting defaults
- Row created callback
- Row grouping
- Footer callback
- Custom toolbar elements
- Order direction sequence
  control

### Styling

- Base style
- Base style - no styling classes
- Base style - cell borders
- Base style - compact
- Base style - hover
- Base style - order-column
- Base style - row borders
- Base style - stripe
- Bootstrap
- Foundation
- jQuery UI ThemeRoller

### Data sources

- HTML (DOM) sourced data
- Ajax sourced data
- Javascript sourced data
- Server-side processing

### API

- Add rows
- Individual column searching (text inputs)
- Individual column searching (select
  inputs)
- Highlighting rows and columns
- Child rows (show extra / detailed
  information)
- Row selection (multiple rows)
- Row selection and deletion (single
  row)
- Form inputs
- Index column
- Show / hide columns dynamically
- Using API in callbacks
- Scrolling and jQuery UI tabs
- Search API (regular expressions)

### Ajax

- Ajax data source (arrays)
- Ajax data source (objects)
- Nested object data (objects)
- Nested object data (arrays)
- Orthogonal data
- Generated content for a column
- Custom data source property
- Flat array data source
- Deferred rendering for speed

### Server-side

- Server-side processing
- Custom HTTP variables
- POST data
- Automatic addition of row ID attributes
- Object data source
- Row details
- Row selection
- JSONP data source for remote domains
- Deferred loading of data
- Pipelining data to reduce Ajax calls for
  paging

### Plug-ins

- API plug-in methods
- Ordering plug-ins (with type
  detection)
- Ordering plug-ins (no type detection)
- Custom filtering - range search
- Live DOM ordering

Please refer to the DataTables documentation for full
information about its API properties and methods.  
Additionally, there are a wide range of extras and
plug-ins which extend the capabilities of
DataTables.

DataTables designed and created by SpryMedia Ltd © 2007-2014  
DataTables is licensed under the MIT license.
