# Supplementary material for: Individual retrotransposon integrants are differentially controlled by KZFP/KAP1-dependent histone methylation, DNA methylation and TET-mediated hydroxymethylation in naïve embryonic stem cells
Source: Epigenetics Chromatin. 2018 Feb 26;11:7. doi: 10.1186/s13072-018-0177-1 (PMC6389204; doi:10.1186/s13072-018-0177-1)
Supplement: Supplementary file 11 — Additional file 11. Pattern analysis. [file 13072_2018_177_MOESM11_ESM.zip › Patterns analysis/DataTables/examples/server_side/index.html]

DataTables examples - Server-side processing


# DataTables example Server-side processing

There are many ways to get your data into DataTables, and if you are working with seriously large
databases, you might want to consider using the server-side options that DataTables provides. With
server-side processing enabled, all paging, searching, ordering etc actions that DataTables performs
are handed off to a server where an SQL engine (or similar) can perform these actions on the large data
set (after all, that's what the database engine is designed for!). As such, each draw of the table will
result in a new Ajax request being made to get the required data.

Server-side processing is enabled by setting the `serverSideDT` option to `true` and
providing an Ajax data source through the `ajaxDT` option.

The examples in this section shows server-side processing in use and how it can be customised to
suit your needs.

### Server-side

- Server-side processing
- Custom HTTP variables
- POST data
- Automatic addition of row ID attributes
- Object data source
- Row details
- Row selection
- JSONP data source for remote domains
- Deferred loading of data
- Pipelining data to reduce Ajax calls for paging

Please refer to the DataTables documentation for full
information about its API properties and methods.  
Additionally, there are a wide range of extras and
plug-ins which extend the capabilities of
DataTables.

DataTables designed and created by SpryMedia Ltd © 2007-2014  
DataTables is licensed under the MIT license.
