# Supplementary material for: Individual retrotransposon integrants are differentially controlled by KZFP/KAP1-dependent histone methylation, DNA methylation and TET-mediated hydroxymethylation in naïve embryonic stem cells
Source: Epigenetics Chromatin. 2018 Feb 26;11:7. doi: 10.1186/s13072-018-0177-1 (PMC6389204; doi:10.1186/s13072-018-0177-1)
Supplement: Supplementary file 11 — Additional file 11. Pattern analysis. [file 13072_2018_177_MOESM11_ESM.zip › Patterns analysis/DataTables/examples/server_side/pipeline.html]

DataTables example - Pipelining data to reduce Ajax calls for paging


# DataTables example Pipelining data to reduce Ajax calls for paging

Sever-side processing can be quite hard on your server, since it makes an Ajax call to the server
for every draw request that is made. On sites with a large number of page views, you could potentially
end up DDoSing your own server with your own applications!

This example shows one technique to reduce the number of Ajax calls that are made to the server by
caching more data than is needed for each draw. This is done by intercepting the Ajax call and routing
it through a data cache control; using the data from the cache if available, and making the Ajax
request if not. This intercept of the Ajax request is performed by giving the `ajaxDT` option as a function. This function
then performs the logic of deciding if another Ajax call is needed, or if data from the cache can be
used.

Keep in mind that this caching is for paging only; the pipeline must be cleared for other
interactions such as ordering and searching since the full data set, when using server-side processing,
is only available at the server.

| First name | Last name | Position | Office | Start date | Salary |
| --- | --- | --- | --- | --- | --- |
| First name | Last name | Position | Office | Start date | Salary |
| --- | --- | --- | --- | --- | --- |

- Javascript
- HTML
- CSS
- Ajax
- Server-side script

The Javascript shown below is used to initialise the table shown in this
example:

`` //
// Pipelining function for DataTables. To be used to the `ajax` option of DataTables
//
$.fn.dataTable.pipeline = function ( opts ) {
// Configuration options
var conf = $.extend( {
pages: 5, // number of pages to cache
url: '', // script url
data: null, // function or object with parameters to send to the server
// matching how `ajax.data` works in DataTables
method: 'GET' // Ajax HTTP method
}, opts );
// Private variables for storing the cache
var cacheLower = -1;
var cacheUpper = null;
var cacheLastRequest = null;
var cacheLastJson = null;
return function ( request, drawCallback, settings ) {
var ajax = false;
var requestStart = request.start;
var drawStart = request.start;
var requestLength = request.length;
var requestEnd = requestStart + requestLength;
if ( settings.clearCache ) {
// API requested that the cache be cleared
ajax = true;
settings.clearCache = false;
}
else if ( cacheLower < 0 || requestStart < cacheLower || requestEnd > cacheUpper ) {
// outside cached data - need to make a request
ajax = true;
}
else if ( JSON.stringify( request.order ) !== JSON.stringify( cacheLastRequest.order ) ||
JSON.stringify( request.columns ) !== JSON.stringify( cacheLastRequest.columns ) ||
JSON.stringify( request.search ) !== JSON.stringify( cacheLastRequest.search )
) {
// properties changed (ordering, columns, searching)
ajax = true;
}
// Store the request for checking next time around
cacheLastRequest = $.extend( true, {}, request );
if ( ajax ) {
// Need data from the server
if ( requestStart < cacheLower ) {
requestStart = requestStart - (requestLength*(conf.pages-1));
if ( requestStart < 0 ) {
requestStart = 0;
}
}
cacheLower = requestStart;
cacheUpper = requestStart + (requestLength * conf.pages);
request.start = requestStart;
request.length = requestLength*conf.pages;
// Provide the same `data` options as DataTables.
if ( $.isFunction ( conf.data ) ) {
// As a function it is executed with the data object as an arg
// for manipulation. If an object is returned, it is used as the
// data object to submit
var d = conf.data( request );
if ( d ) {
$.extend( request, d );
}
}
else if ( $.isPlainObject( conf.data ) ) {
// As an object, the data given extends the default
$.extend( request, conf.data );
}
settings.jqXHR = $.ajax( {
"type": conf.method,
"url": conf.url,
"data": request,
"dataType": "json",
"cache": false,
"success": function ( json ) {
cacheLastJson = $.extend(true, {}, json);
if ( cacheLower != drawStart ) {
json.data.splice( 0, drawStart-cacheLower );
}
json.data.splice( requestLength, json.data.length );
drawCallback( json );
}
} );
}
else {
json = $.extend( true, {}, cacheLastJson );
json.draw = request.draw; // Update the echo for each response
json.data.splice( 0, requestStart-cacheLower );
json.data.splice( requestLength, json.data.length );
drawCallback(json);
}
}
};
// Register an API method that will empty the pipelined data, forcing an Ajax
// fetch on the next draw (i.e. `table.clearPipeline().draw()`)
$.fn.dataTable.Api.register( 'clearPipeline()', function () {
return this.iterator( 'table', function ( settings ) {
settings.clearCache = true;
} );
} );
//
// DataTables initialisation
//
$(document).ready(function() {
$('#example').dataTable( {
"processing": true,
"serverSide": true,
"ajax": $.fn.dataTable.pipeline( {
url: 'scripts/server_processing.php',
pages: 5 // number of pages to cache
} )
} );
} ); ``

In addition to the above code, the following Javascript library files are loaded for use in this
example:

- ../../media/js/jquery.js
- ../../media/js/jquery.dataTables.js

The HTML shown below is the raw HTML table element, before it has been enhanced by
DataTables:

This example uses a little bit of additional CSS beyond what is loaded from the library
files (below), in order to correctly display the table. The additional CSS used is shown
below:

The following CSS library files are loaded for use in this example to provide the styling of the
table:

- ../../media/css/jquery.dataTables.css

This table loads data by Ajax. The latest data that has been loaded is shown below. This data
will update automatically as any additional data is loaded.

The script used to perform the server-side processing for this table is shown below. Please note
that this is just an example script using PHP. Server-side processing scripts can be written in any
language, using the protocol described in the
DataTables documentation.

## Other examples

### Basic initialisation

- Zero configuration
- Feature enable / disable
- Default ordering (sorting)
- Multi-column ordering
- Multiple tables
- Hidden columns
- Complex headers (rowspan and
  colspan)
- DOM positioning
- Flexible table width
- State saving
- Alternative pagination
- Scroll - vertical
- Scroll - horizontal
- Scroll - horizontal and vertical
- Scroll - vertical with jQuery UI
  ThemeRoller
- Language - Comma decimal place
- Language options

### Advanced initialisation

- DOM / jQuery events
- DataTables events
- Column rendering
- Page length options
- Multiple table control
  elements
- Complex headers (rowspan /
  colspan)
- Read HTML to data objects
- HTML5 data-\* attributes
- Language file
- Setting defaults
- Row created callback
- Row grouping
- Footer callback
- Custom toolbar elements
- Order direction sequence
  control

### Styling

- Base style
- Base style - no styling classes
- Base style - cell borders
- Base style - compact
- Base style - hover
- Base style - order-column
- Base style - row borders
- Base style - stripe
- Bootstrap
- Foundation
- jQuery UI ThemeRoller

### Data sources

- HTML (DOM) sourced data
- Ajax sourced data
- Javascript sourced data
- Server-side processing

### API

- Add rows
- Individual column searching (text inputs)
- Individual column searching (select
  inputs)
- Highlighting rows and columns
- Child rows (show extra / detailed
  information)
- Row selection (multiple rows)
- Row selection and deletion (single
  row)
- Form inputs
- Index column
- Show / hide columns dynamically
- Using API in callbacks
- Scrolling and jQuery UI tabs
- Search API (regular expressions)

### Ajax

- Ajax data source (arrays)
- Ajax data source (objects)
- Nested object data (objects)
- Nested object data (arrays)
- Orthogonal data
- Generated content for a column
- Custom data source property
- Flat array data source
- Deferred rendering for speed

### Server-side

- Server-side processing
- Custom HTTP variables
- POST data
- Automatic addition of row ID attributes
- Object data source
- Row details
- Row selection
- JSONP data source for remote domains
- Deferred loading of data
- Pipelining data to reduce Ajax calls for
  paging

### Plug-ins

- API plug-in methods
- Ordering plug-ins (with type
  detection)
- Ordering plug-ins (no type
  detection)
- Custom filtering - range search
- Live DOM ordering

Please refer to the DataTables documentation for full
information about its API properties and methods.  
Additionally, there are a wide range of extras and
plug-ins which extend the capabilities of
DataTables.

DataTables designed and created by SpryMedia Ltd © 2007-2014  
DataTables is licensed under the MIT license.
