# Supplementary material for: Individual retrotransposon integrants are differentially controlled by KZFP/KAP1-dependent histone methylation, DNA methylation and TET-mediated hydroxymethylation in naïve embryonic stem cells
Source: Epigenetics Chromatin. 2018 Feb 26;11:7. doi: 10.1186/s13072-018-0177-1 (PMC6389204; doi:10.1186/s13072-018-0177-1)
Supplement: Supplementary file 11 — Additional file 11. Pattern analysis. [file 13072_2018_177_MOESM11_ESM.zip › Patterns analysis/DataTables/examples/server_side/row_details.html]

DataTables example - Row details


# DataTables example Row details

This example shows the use of DataTables' ability to show and hide child rows which are attached to
a parent row in the host table. This is often used to show additional information about a row,
particularly when you wish to convey more information about a row than there is space for in the host
table.

The example below shows server-side processing being used with the first column having an event
listener attached to it which will toggle the child row's display. This is set up using `columns.dataDT` and `columns.defaultContentDT`, in combination
with CSS to show an empty cell with a background image which can be clicked upon.

The event handler makes use of the `row().childDT` methods to firstly check if a
row is already displayed, and if so hide it, if not show it. The content of the child row is, in this
example, defined by the `formatDetails()` function, but you would replace that with whatever
you wanted to show the content required, possibly including, for example, an Ajax call to the server to
obtain the extra information to show. Note that the format details function has access to the full data
source object for the row, including information that is not actually shown in the table (the salary
parameter for example).

Furthermore, this example shows a small difference from the client-side row details example in that to have rows automatically reopen
when the table is redrawn, we need to track a unique identifier for each row - in this case the row
`id`. This is required because in server-side processing mode rows are automatically
destroyed and recreated on each draw.

|  | First name | Last name | Position | Office |
| --- | --- | --- | --- | --- |
|  | First name | Last name | Position | Office |
| --- | --- | --- | --- | --- |

- Javascript
- HTML
- CSS
- Ajax
- Server-side script

The Javascript shown below is used to initialise the table shown in this
example:

`` function format ( d ) {
return 'Full name: '+d.first_name+' '+d.last_name+'<br>'+
'Salary: '+d.salary+'<br>'+
'The child row can contain any data you wish, including links, images, inner tables etc.';
}
$(document).ready(function() {
var dt = $('#example').DataTable( {
"processing": true,
"serverSide": true,
"ajax": "scripts/ids-objects.php",
"columns": [
{
"class": "details-control",
"orderable": false,
"data": null,
"defaultContent": ""
},
{ "data": "first_name" },
{ "data": "last_name" },
{ "data": "position" },
{ "data": "office" }
],
"order": [[1, 'asc']]
} );
// Array to track the ids of the details displayed rows
var detailRows = [];
$('#example tbody').on( 'click', 'tr td:first-child', function () {
var tr = $(this).closest('tr');
var row = dt.row( tr );
var idx = $.inArray( tr.attr('id'), detailRows );
if ( row.child.isShown() ) {
tr.removeClass( 'details' );
row.child.hide();
// Remove from the 'open' array
detailRows.splice( idx, 1 );
}
else {
tr.addClass( 'details' );
row.child( format( row.data() ) ).show();
// Add to the 'open' array
if ( idx === -1 ) {
detailRows.push( tr.attr('id') );
}
}
} );
// On each draw, loop over the `detailRows` array and show any child rows
dt.on( 'draw', function () {
$.each( detailRows, function ( i, id ) {
$('#'+id+' td:first-child').trigger( 'click' );
} );
} );
} ); ``

In addition to the above code, the following Javascript library files are loaded for use in this
example:

- ../../media/js/jquery.js
- ../../media/js/jquery.dataTables.js

The HTML shown below is the raw HTML table element, before it has been enhanced by
DataTables:

This example uses a little bit of additional CSS beyond what is loaded from the library
files (below), in order to correctly display the table. The additional CSS used is shown
below:

`td.details-control {
background: url('../resources/details_open.png') no-repeat center center;
cursor: pointer;
}
tr.details td.details-control {
background: url('../resources/details_close.png') no-repeat center center;
}`

The following CSS library files are loaded for use in this example to provide the styling of the
table:

- ../../media/css/jquery.dataTables.css

This table loads data by Ajax. The latest data that has been loaded is shown below. This data
will update automatically as any additional data is loaded.

The script used to perform the server-side processing for this table is shown below. Please note
that this is just an example script using PHP. Server-side processing scripts can be written in any
language, using the protocol described in the
DataTables documentation.

## Other examples

### Basic initialisation

- Zero configuration
- Feature enable / disable
- Default ordering (sorting)
- Multi-column ordering
- Multiple tables
- Hidden columns
- Complex headers (rowspan and
  colspan)
- DOM positioning
- Flexible table width
- State saving
- Alternative pagination
- Scroll - vertical
- Scroll - horizontal
- Scroll - horizontal and vertical
- Scroll - vertical with jQuery UI
  ThemeRoller
- Language - Comma decimal place
- Language options

### Advanced initialisation

- DOM / jQuery events
- DataTables events
- Column rendering
- Page length options
- Multiple table control
  elements
- Complex headers (rowspan /
  colspan)
- Read HTML to data objects
- HTML5 data-\* attributes
- Language file
- Setting defaults
- Row created callback
- Row grouping
- Footer callback
- Custom toolbar elements
- Order direction sequence
  control

### Styling

- Base style
- Base style - no styling classes
- Base style - cell borders
- Base style - compact
- Base style - hover
- Base style - order-column
- Base style - row borders
- Base style - stripe
- Bootstrap
- Foundation
- jQuery UI ThemeRoller

### Data sources

- HTML (DOM) sourced data
- Ajax sourced data
- Javascript sourced data
- Server-side processing

### API

- Add rows
- Individual column searching (text inputs)
- Individual column searching (select
  inputs)
- Highlighting rows and columns
- Child rows (show extra / detailed
  information)
- Row selection (multiple rows)
- Row selection and deletion (single
  row)
- Form inputs
- Index column
- Show / hide columns dynamically
- Using API in callbacks
- Scrolling and jQuery UI tabs
- Search API (regular expressions)

### Ajax

- Ajax data source (arrays)
- Ajax data source (objects)
- Nested object data (objects)
- Nested object data (arrays)
- Orthogonal data
- Generated content for a column
- Custom data source property
- Flat array data source
- Deferred rendering for speed

### Server-side

- Server-side processing
- Custom HTTP variables
- POST data
- Automatic addition of row ID attributes
- Object data source
- Row details
- Row selection
- JSONP data source for remote domains
- Deferred loading of data
- Pipelining data to reduce Ajax calls for paging

### Plug-ins

- API plug-in methods
- Ordering plug-ins (with type
  detection)
- Ordering plug-ins (no type
  detection)
- Custom filtering - range search
- Live DOM ordering

Please refer to the DataTables documentation for full
information about its API properties and methods.  
Additionally, there are a wide range of extras and
plug-ins which extend the capabilities of
DataTables.

DataTables designed and created by SpryMedia Ltd © 2007-2014  
DataTables is licensed under the MIT license.
