# Supplementary material for: Individual retrotransposon integrants are differentially controlled by KZFP/KAP1-dependent histone methylation, DNA methylation and TET-mediated hydroxymethylation in naïve embryonic stem cells
Source: Epigenetics Chromatin. 2018 Feb 26;11:7. doi: 10.1186/s13072-018-0177-1 (PMC6389204; doi:10.1186/s13072-018-0177-1)
Supplement: Supplementary file 11 — Additional file 11. Pattern analysis. [file 13072_2018_177_MOESM11_ESM.zip › Patterns analysis/DataTables/examples/styling/index.html]

DataTables examples - Styling


# DataTables example Styling

When using DataTables, you want your tables to fit in with your site / app to make the end user
experience as seamless as possible. For this reason, DataTables provides an easy to customise core
stylesheet, which has a number of features that you can enable or disable as you required. Additionally
there are a number of integration packages which can be used to fit DataTables into a site which uses
some of the popular CSS libraries such as Twitter Bootstrap and Foundation. Or of course you can craft
your own CSS to fit it into your site perfectly!

This section includes examples of how DataTables can be styled using these methods.

### Styling

- Base style
- Base style - no styling classes
- Base style - cell borders
- Base style - compact
- Base style - hover
- Base style - order-column
- Base style - row borders
- Base style - stripe
- Bootstrap
- Foundation
- jQuery UI ThemeRoller

Please refer to the DataTables documentation for full
information about its API properties and methods.  
Additionally, there are a wide range of extras and
plug-ins which extend the capabilities of
DataTables.

DataTables designed and created by SpryMedia Ltd © 2007-2014  
DataTables is licensed under the MIT license.
