# Supplementary material for: Individual retrotransposon integrants are differentially controlled by KZFP/KAP1-dependent histone methylation, DNA methylation and TET-mediated hydroxymethylation in naïve embryonic stem cells
Source: Epigenetics Chromatin. 2018 Feb 26;11:7. doi: 10.1186/s13072-018-0177-1 (PMC6389204; doi:10.1186/s13072-018-0177-1)
Supplement: Supplementary file 11 — Additional file 11. Pattern analysis. [file 13072_2018_177_MOESM11_ESM.zip › Patterns analysis/DataTables/extensions/AutoFill/examples/index.html]

AutoFill examples - AutoFill examples


# AutoFill example AutoFill examples

AutoFill gives an Excel like option to a DataTable to click and drag over multiple cells, filling in
information over the selected cells and incrementing numbers as needed.

Thanks to Phoniax AS for their sponsorship of this plug-in for
DataTables.

### Examples

- Basic initialisation
- Column options
- Scrolling DataTable
- Horizontal and vertical fill
- Horizontal fill
- Complete callback
- Step callback

Please refer to the DataTables documentation for full
information about its API properties and methods.  
Additionally, there are a wide range of extras and
plug-ins which extend the capabilities of
DataTables.

DataTables designed and created by SpryMedia Ltd © 2007-2014  
DataTables is licensed under the MIT license.
