# Supplementary material for: Individual retrotransposon integrants are differentially controlled by KZFP/KAP1-dependent histone methylation, DNA methylation and TET-mediated hydroxymethylation in naïve embryonic stem cells
Source: Epigenetics Chromatin. 2018 Feb 26;11:7. doi: 10.1186/s13072-018-0177-1 (PMC6389204; doi:10.1186/s13072-018-0177-1)
Supplement: Supplementary file 11 — Additional file 11. Pattern analysis. [file 13072_2018_177_MOESM11_ESM.zip › Patterns analysis/DataTables/extensions/ColReorder/examples/alt_insert.html]

ColReorder example - Alternative insert styling


# ColReorder example Alternative insert styling

Using CSS it is easy to modify the insert bar to suit your web-site. This example shows how an arrow
can be used to show the insert point rather than the straight bar used in the other examples by simply
adding an extra CSS rule to include the image.

| Name | Position | Office | Age | Start date | Salary |
| --- | --- | --- | --- | --- | --- |
| Name | Position | Office | Age | Start date | Salary |
| --- | --- | --- | --- | --- | --- |
| Tiger Nixon | System Architect | Edinburgh | 61 | 2011/04/25 | $320,800 |
| Garrett Winters | Accountant | Tokyo | 63 | 2011/07/25 | $170,750 |
| Ashton Cox | Junior Technical Author | San Francisco | 66 | 2009/01/12 | $86,000 |
| Cedric Kelly | Senior Javascript Developer | Edinburgh | 22 | 2012/03/29 | $433,060 |
| Airi Satou | Accountant | Tokyo | 33 | 2008/11/28 | $162,700 |
| Brielle Williamson | Integration Specialist | New York | 61 | 2012/12/02 | $372,000 |
| Herrod Chandler | Sales Assistant | San Francisco | 59 | 2012/08/06 | $137,500 |
| Rhona Davidson | Integration Specialist | Tokyo | 55 | 2010/10/14 | $327,900 |
| Colleen Hurst | Javascript Developer | San Francisco | 39 | 2009/09/15 | $205,500 |
| Sonya Frost | Software Engineer | Edinburgh | 23 | 2008/12/13 | $103,600 |
| Jena Gaines | Office Manager | London | 30 | 2008/12/19 | $90,560 |
| Quinn Flynn | Support Lead | Edinburgh | 22 | 2013/03/03 | $342,000 |
| Charde Marshall | Regional Director | San Francisco | 36 | 2008/10/16 | $470,600 |
| Haley Kennedy | Senior Marketing Designer | London | 43 | 2012/12/18 | $313,500 |
| Tatyana Fitzpatrick | Regional Director | London | 19 | 2010/03/17 | $385,750 |
| Michael Silva | Marketing Designer | London | 66 | 2012/11/27 | $198,500 |
| Paul Byrd | Chief Financial Officer (CFO) | New York | 64 | 2010/06/09 | $725,000 |
| Gloria Little | Systems Administrator | New York | 59 | 2009/04/10 | $237,500 |
| Bradley Greer | Software Engineer | London | 41 | 2012/10/13 | $132,000 |
| Dai Rios | Personnel Lead | Edinburgh | 35 | 2012/09/26 | $217,500 |
| Jenette Caldwell | Development Lead | New York | 30 | 2011/09/03 | $345,000 |
| Yuri Berry | Chief Marketing Officer (CMO) | New York | 40 | 2009/06/25 | $675,000 |
| Caesar Vance | Pre-Sales Support | New York | 21 | 2011/12/12 | $106,450 |
| Doris Wilder | Sales Assistant | Sidney | 23 | 2010/09/20 | $85,600 |
| Angelica Ramos | Chief Executive Officer (CEO) | London | 47 | 2009/10/09 | $1,200,000 |
| Gavin Joyce | Developer | Edinburgh | 42 | 2010/12/22 | $92,575 |
| Jennifer Chang | Regional Director | Singapore | 28 | 2010/11/14 | $357,650 |
| Brenden Wagner | Software Engineer | San Francisco | 28 | 2011/06/07 | $206,850 |
| Fiona Green | Chief Operating Officer (COO) | San Francisco | 48 | 2010/03/11 | $850,000 |
| Shou Itou | Regional Marketing | Tokyo | 20 | 2011/08/14 | $163,000 |
| Michelle House | Integration Specialist | Sidney | 37 | 2011/06/02 | $95,400 |
| Suki Burks | Developer | London | 53 | 2009/10/22 | $114,500 |
| Prescott Bartlett | Technical Author | London | 27 | 2011/05/07 | $145,000 |
| Gavin Cortez | Team Leader | San Francisco | 22 | 2008/10/26 | $235,500 |
| Martena Mccray | Post-Sales support | Edinburgh | 46 | 2011/03/09 | $324,050 |
| Unity Butler | Marketing Designer | San Francisco | 47 | 2009/12/09 | $85,675 |
| Howard Hatfield | Office Manager | San Francisco | 51 | 2008/12/16 | $164,500 |
| Hope Fuentes | Secretary | San Francisco | 41 | 2010/02/12 | $109,850 |
| Vivian Harrell | Financial Controller | San Francisco | 62 | 2009/02/14 | $452,500 |
| Timothy Mooney | Office Manager | London | 37 | 2008/12/11 | $136,200 |
| Jackson Bradshaw | Director | New York | 65 | 2008/09/26 | $645,750 |
| Olivia Liang | Support Engineer | Singapore | 64 | 2011/02/03 | $234,500 |
| Bruno Nash | Software Engineer | London | 38 | 2011/05/03 | $163,500 |
| Sakura Yamamoto | Support Engineer | Tokyo | 37 | 2009/08/19 | $139,575 |
| Thor Walton | Developer | New York | 61 | 2013/08/11 | $98,540 |
| Finn Camacho | Support Engineer | San Francisco | 47 | 2009/07/07 | $87,500 |
| Serge Baldwin | Data Coordinator | Singapore | 64 | 2012/04/09 | $138,575 |
| Zenaida Frank | Software Engineer | New York | 63 | 2010/01/04 | $125,250 |
| Zorita Serrano | Software Engineer | San Francisco | 56 | 2012/06/01 | $115,000 |
| Jennifer Acosta | Junior Javascript Developer | Edinburgh | 43 | 2013/02/01 | $75,650 |
| Cara Stevens | Sales Assistant | New York | 46 | 2011/12/06 | $145,600 |
| Hermione Butler | Regional Director | London | 47 | 2011/03/21 | $356,250 |
| Lael Greer | Systems Administrator | London | 21 | 2009/02/27 | $103,500 |
| Jonas Alexander | Developer | San Francisco | 30 | 2010/07/14 | $86,500 |
| Shad Decker | Regional Director | Edinburgh | 51 | 2008/11/13 | $183,000 |
| Michael Bruce | Javascript Developer | Singapore | 29 | 2011/06/27 | $183,000 |
| Donna Snider | Customer Support | New York | 27 | 2011/01/25 | $112,000 |

- Javascript
- HTML
- CSS
- Ajax
- Server-side script

The Javascript shown below is used to initialise the table shown in this
example:

`$(document).ready(function() {
$('#example').DataTable( {
dom: 'Rlfrtip'
} );
} );`

In addition to the above code, the following Javascript library files are loaded for use in this
example:

- ../../../media/js/jquery.js
- ../../../media/js/jquery.dataTables.js
- ../js/dataTables.colReorder.js

The HTML shown below is the raw HTML table element, before it has been enhanced by
DataTables:

This example uses a little bit of additional CSS beyond what is loaded from the library
files (below), in order to correctly display the table. The additional CSS used is shown
below:

`div.DTCR_pointer {
margin-top: -15px;
margin-left: -9px;
width: 18px;
background: url('../images/insert.png') no-repeat top left;
}`

The following CSS library files are loaded for use in this example to provide the styling of the
table:

- ../../../media/css/jquery.dataTables.css
- ../css/dataTables.colReorder.css

This table loads data by Ajax. The latest data that has been loaded is shown below. This data
will update automatically as any additional data is loaded.

The script used to perform the server-side processing for this table is shown below. Please note
that this is just an example script using PHP. Server-side processing scripts can be written in any
language, using the protocol described in the
DataTables documentation.

## Other examples

### Examples

- Basic initialisation
- Initialisation using `new`
- Alternative insert styling
- Realtime updating
- State saving
- Scrolling table
- Predefined column ordering
- Reset ordering API
- ColVis integration
- FixedColumns integration
- FixedHeader integration
- jQuery UI styling
- Individual column filtering
- Server-side processing

Please refer to the DataTables documentation for full
information about its API properties and methods.  
Additionally, there are a wide range of extras and
plug-ins which extend the capabilities of
DataTables.

DataTables designed and created by SpryMedia Ltd © 2007-2014  
DataTables is licensed under the MIT license.
