# Supplementary material for: Individual retrotransposon integrants are differentially controlled by KZFP/KAP1-dependent histone methylation, DNA methylation and TET-mediated hydroxymethylation in naïve embryonic stem cells
Source: Epigenetics Chromatin. 2018 Feb 26;11:7. doi: 10.1186/s13072-018-0177-1 (PMC6389204; doi:10.1186/s13072-018-0177-1)
Supplement: Supplementary file 11 — Additional file 11. Pattern analysis. [file 13072_2018_177_MOESM11_ESM.zip › Patterns analysis/DataTables/extensions/ColVis/examples/two_tables.html]

ColVis example - Two tables with individual controls


# ColVis example Two tables with individual controls

It can be useful to have DataTables initialise more than one table with a single call can for them
to each have individual ColVis controllers. All this requires is a suitable jQuery selector to be used,
and DataTables and ColVis will take care of the rest - as shown in this example.

| Name | Position | Office | Age | Salary |
| --- | --- | --- | --- | --- |
| Name | Position | Office | Age | Salary |
| --- | --- | --- | --- | --- |
| Tiger Nixon | System Architect | Edinburgh | 61 | $320,800 |
| Cedric Kelly | Senior Javascript Developer | Edinburgh | 22 | $433,060 |
| Sonya Frost | Software Engineer | Edinburgh | 23 | $103,600 |
| Quinn Flynn | Support Lead | Edinburgh | 22 | $342,000 |
| Dai Rios | Personnel Lead | Edinburgh | 35 | $217,500 |
| Gavin Joyce | Developer | Edinburgh | 42 | $92,575 |
| Martena Mccray | Post-Sales support | Edinburgh | 46 | $324,050 |
| Jennifer Acosta | Junior Javascript Developer | Edinburgh | 43 | $75,650 |
| Shad Decker | Regional Director | Edinburgh | 51 | $183,000 |

| Name | Position | Office | Age | Salary |
| --- | --- | --- | --- | --- |
| Name | Position | Office | Age | Salary |
| --- | --- | --- | --- | --- |
| Jena Gaines | Office Manager | London | 30 | $90,560 |
| Haley Kennedy | Senior Marketing Designer | London | 43 | $313,500 |
| Tatyana Fitzpatrick | Regional Director | London | 19 | $385,750 |
| Michael Silva | Marketing Designer | London | 66 | $198,500 |
| Bradley Greer | Software Engineer | London | 41 | $132,000 |
| Angelica Ramos | Chief Executive Officer (CEO) | London | 47 | $1,200,000 |
| Suki Burks | Developer | London | 53 | $114,500 |
| Prescott Bartlett | Technical Author | London | 27 | $145,000 |
| Timothy Mooney | Office Manager | London | 37 | $136,200 |
| Bruno Nash | Software Engineer | London | 38 | $163,500 |
| Hermione Butler | Regional Director | London | 47 | $356,250 |
| Lael Greer | Systems Administrator | London | 21 | $103,500 |

- Javascript
- HTML
- CSS
- Ajax
- Server-side script

The Javascript shown below is used to initialise the table shown in this
example:

`$(document).ready(function() {
$('table.display').DataTable( {
dom: 'C<"clear">lfrtip',
displayLength: 5
} );
} );`

In addition to the above code, the following Javascript library files are loaded for use in this
example:

- ../../../media/js/jquery.js
- ../../../media/js/jquery.dataTables.js
- ../js/dataTables.colVis.js

The HTML shown below is the raw HTML table element, before it has been enhanced by
DataTables:

This example uses a little bit of additional CSS beyond what is loaded from the library
files (below), in order to correctly display the table. The additional CSS used is shown
below:

The following CSS library files are loaded for use in this example to provide the styling of the
table:

- ../../../media/css/jquery.dataTables.css
- ../css/dataTables.colVis.css

This table loads data by Ajax. The latest data that has been loaded is shown below. This data
will update automatically as any additional data is loaded.

The script used to perform the server-side processing for this table is shown below. Please note
that this is just an example script using PHP. Server-side processing scripts can be written in any
language, using the protocol described in the
DataTables documentation.

## Other examples

### Examples

- Basic initialisation
- `new` initialisation
- Custom button text
- Exclude columns from list
- Column button callback
- Button ordering
- Mouseover activation
- Group columns
- Two tables with individual controls
- Two tables with shared controls
- Restore / show all
- jQuery UI styling

Please refer to the DataTables documentation for full
information about its API properties and methods.  
Additionally, there are a wide range of extras and
plug-ins which extend the capabilities of
DataTables.

DataTables designed and created by SpryMedia Ltd © 2007-2014  
DataTables is licensed under the MIT license.
